# A panel of eight microRNAs is a good predictive parameter for triple-negative breast cancer relapse

**DOI:** 10.7150/thno.46142

**Published:** 2020-07-09

**Authors:** Hsiao-Chin Hong, Cheng-Hsun Chuang, Wei-Chih Huang, Shun-Long Weng, Chia-Hung Chen, Kuang-Hsin Chang, Kuang-Wen Liao, Hsien-Da Huang

**Affiliations:** 1 Warshel Institute for Computational Biology, The Chinese University of Hong Kong, Shenzhen, Guangdong Province 518172, China; 2 School of Life and Health Sciences, The Chinese University of Hong Kong, Shenzhen, Guangdong Province 518172, China; 3 Institute of Molecular Medicine and Bioengineering, National Chiao Tung University, Hsinchu City 30068, Taiwan, ROC; 4 Institute of Bioinformatics and Systems Biology, National Chiao Tung University, Hsinchu City 30068, Taiwan, ROC; 5 Department of Biological Science and Technology, National Chiao Tung University, Hsinchu City 30068, Taiwan, ROC; 6 Come True Biomedical Inc., Taichung 408, Taiwan, ROC; 7 Department of Obstetrics and Gynecology, Hsinchu MacKay Memorial Hospital, Hsinchu City 300, Taiwan, ROC; 8 Department of Medicine, MacKay Medical College, New Taipei City 252, Taiwan, ROC; 9 MacKay Junior College of Medicine, Nursing and Management College, Taipei City 112, Taiwan, ROC; 10 Department of Medical Research, Hsinchu Mackay Memorial Hospital, Hsinchu City 30071, Taiwan, ROC; 11 Center for Intelligent Drug Systems and Smart Bio-Devices, National Chiao Tung University, Hsinchu City 30068, Taiwan, ROC; 12 Graduate Institute of Medicine, College of Medicine, Kaohsiung Medical University, Kaohsiung 80708, Taiwan, ROC

**Keywords:** triple-negative breast cancer, miRNA signature, relapse, prediction, prognosis

## Abstract

**Rationale:** Triple-negative breast cancer (TNBC), which has the highest recurrence rate and shortest survival time of all breast cancers, is in urgent need of a risk assessment method to determine an accurate treatment course. Recently, miRNA expression patterns have been identified as potential biomarkers for diagnosis, prognosis, and personalized therapy. Here, we investigate a combination of candidate miRNAs as a clinically applicable signature that can precisely predict relapse in TNBC patients after surgery.

**Methods:** Four total cohorts of training (TCGA_TNBC and GEOD-40525) and validation (GSE40049 and GSE19783) datasets were analyzed with logistic regression and Gaussian mixture analyses. We established a miRNA signature risk model and identified an 8-miRNA signature for the prediction of TNBC relapse.

**Results:** The miRNA signature risk model identified ten candidate miRNAs in the training set. By combining 8 of the 10 miRNAs (miR-139-5p, miR-10b-5p, miR-486-5p, miR-455-3p, miR-107, miR-146b-5p, miR-324-5p and miR-20a-5p), an accurate predictive model of relapse in TNBC patients was established and was highly correlated with prognosis (AUC of 0.80). Subsequently, this 8-miRNA signature prognosticated relapse in the two validation sets with AUCs of 0.89 and 0.90.

**Conclusion:** The 8-miRNA signature predictive model may help clinicians provide a prognosis for TNBC patients with a high risk of recurrence after surgery and provide further personalized treatment to decrease the chance of relapse.

## Introduction

Breast cancer (BC) is one of the most common causes of death in women worldwide 1, 2. BC is not a single disease and is composed of several subtypes, such as luminal A, luminal B, HER2 and triple-negative breast cancer (TNBC). TNBC does not express or expresses low levels of the estrogen receptor (ER), progesterone receptor (PR) and human epidermal growth factor receptor 2 (HER2). TNBC occurs in approximately 10-20% of patients diagnosed with BC at a young age (40-50 years old). TNBC is an advanced multidrug resistant (MDR) breast cancer with a high recurrence rate within the first three to five years and a short overall survival (OS) rate 3, 4. The causes behind survival differences are diverse, including genetic predispositions, lifestyle and other environmental factors 5-7. Currently, the treatment strategies for TNBC are limited to surgery, chemotherapy, and radiation owing to the lack of effective therapeutic targets. Moreover, due to the high tumor heterogeneity, there is a lack of definitive clinical determinants in TNBC-specific diagnostic or prognostic markers 8.

MicroRNAs (miRNAs) are small noncoding RNAs that are 18-25 nucleotides in length and negatively regulate gene expression by translational repression or mRNA degradation. Previous evidence has demonstrated that miRNAs facilitate tumor growth, migration, invasion, and angiogenesis as well as cell survival and immune evasion by targeting mRNAs 9, 10. In addition, many studies have reported that miRNAs may function as potential diagnostic and prognostic biomarkers for different cancers 11. Dominika Piasecka et al. found that upregulation of miR-10b, miR-21, miR-29, miR-221/222, and miR-373 and downregulation of miR-145, miR-199a-5p, miR-200 family members, miR-203, and miR-205 were significantly associated with epithelial-to-mesenchymal transition (EMT) or cancer stem cell (CSC)-like properties and have prognostic value in TNBC patients 12, 13.

In the field of oncology, biomarkers generally possess three types of clinical relevance: diagnostic value, prognostic value, and predictive value. The prognostic value includes the prediction of disease outcomes or risk assessments independent of treatment 14. The predictive value involves the prediction of responses to treatments as well as sensitive and specific biomarkers of clinical outcomes at a relatively early stage. Moreover, the integration of biomarker data using bioinformatics methods will enhance our understanding of biological pathways and regulatory mechanisms associated with diseases. Next-generation sequencing (NGS) and microarrays have increasingly been used to measure the expression levels of miRNAs. Advanced bioinformatics analysis methods with high efficiency, sensitivity and specificity play essential roles in miRNA biomarker development 15, 16.

The tumor-node-metastasis (TNM) staging system is a classification system based on the characteristics of the tumor, regional lymph nodes, and metastatic sites. In addition, it correlates important tumor characteristics with survival data to help estimate and follow outcomes 17. However, the current TNM staging system is inadequate for identifying high-risk patients. To resolve this problem, we conducted an extensive miRNA profiling study on TNBC patients with public datasets. Each tumor type presents with a unique miRNA signature, which can be used to identify new diagnoses, prognoses and potential biomarkers for personalized medicine 18, 19. Using systemic and comprehensive bioinformatics methods to train and validate the approach, we aimed to identify an 8-miRNA signature that can improve the current TNM staging system and that is superior to the currently offered molecular assays to predict relapse in TNBC patients after surgery. Moreover, this signature may have clinical implications in the molecular biomarkers of different cancers, development of targeted therapy, or selection of high-risk cancer patients for adjuvant chemotherapy 20, 21.

## Methods

### Collection and processing of expression profile data

Two public datasets were analyzed in the training set: TNBC miRNA sequencing data from TCGA_BRCA level 3 data (The Cancer Genome Atlas (TCGA, https://www.ncbi.nlm.nih.gov/) and GEOD-40525 data from Gene Expression Omnibus (GEO, https://www.ncbi.nlm.nih.gov/gds). All datasets followed the classification system of Voduc et al, which is based on the immunohistochemical (IHC) semiquantitative analysis of ER, PR and HER2 expression, as recommended by international guidelines 22. The TCGA_BRCA data had 117 TNBC (TCGA_TNBC dataset) and 637 non-TNBC (TCGA_non-TNBC dataset) patients. The TCGA_TNBC and GEOD-40525 datasets include 125 patients with corresponding miRNA sequencing data derived from two different platforms. The TCGA_TNBC dataset was obtained through Illumina HiSeq 2000 miRNA sequencing (n=117). The miRNA expression levels, measured by reads per million miRNAs mapped (RPM), were first log2 transformed. The GEOD-40525 dataset was based on an Agilent-019118 Human miRNA Microarray 2.0 platform (n=8). The top 10 miRNAs (miR-139-5p, miR-10b-5p, miR-486-5p, miR-455-3p, miR-107, miR-146b-5p, miR-17-5p, miR-324-5p, miR-20a-5p and miR-142-3p) were identified after adjustment for multiple comparisons: *p*-value<0.05 and FDR <0.05. The validation set contained three public datasets, GSE40049, GSE19783 and E-MTAB-1989, from Applied Biosystems SOLiD sequencing (n=24), an Agilent-019118 Human miRNA Microarray 2.0 (n=18) platform and an Affymetrix GeneChip miRNA 2.0 Array (n=18), respectively. The validation data were from GEO (https://www.ncbi.nlm.nih.gov/gds) and ArrayExpress (https://www.ebi.ac.uk/arrayexpress).

### Gaussian mixture and logistic regression models for predicting recurrence

Classification was conducted with model-based hierarchical agglomerative clustering based on the Gaussian finite mixture model. The miRNA clusters were classified by the Gaussian mixture model (GMM). Logistic regression analysis was used to construct combined models to predict recurrence. Receiver operating characteristic (ROC) curves were constructed to assess the predictive value of the models by calculating the AUCs. With the predictive miRNA signature model, the risk scores for the 111 TNBC patients were calculated from the TCGA_TNBC dataset. The TNBC patients were classified into recurrence and nonrecurrence groups using the median risk score as the cutoff value. The sensitivity and specificity of the miRNA prognostic signature to predict clinical outcome was evaluated by calculating the AUC value of the ROC curve using an R package. The associations between disease-free survival (DFS) and OS miRNA expression levels were estimated by the Kaplan-Meier method, log-rank test (Mantel-Cox) and Gehan-Breslow-Wilcoxon methods. Differences in survival between the high expression and the low expression miRNAs were analyzed using the two-sided log-rank test.

### MiRNA-target interactions (MTIs)

miRTarBase 7.0 is a comprehensive collection of MTIs that have been experimentally validated 23. The biological features of miRNA/target duplexes are assessed based on the largest collection of MTIs currently available. miRTarBase uses a pipeline combining text-mining and manual review methods.

### Functional analysis

Gene set enrichment analysis (GSEA) was performed by using the software provided by the Broad Institute. Functional enrichment was achieved with MSigDB and the GSEA method. In this study, we found the top 20 biological functions and pathways by using the R packages ggplot2, clusterProfiler 24 and DOSE 25 for the statistical analysis of Gene Ontology (GO) and Hallmark gene sets in the gene clusters. The Reactome knowledgebase provides molecular details of signal transduction, transport, DNA replication, metabolism, and other cellular processes as an ordered network of molecular transformations and is an extended version of a classic metabolic map in a single consistent data model.

### Statistical analyses

All statistical analyses were performed using R software (version 3.5.1), the mclust R package 26, the pROC package version 1.8 and GraphPad Prism versions 6 and 8 (San Diego, California USA). Venny 2.1 and GENE-E were used to determine the distribution of the differentially expressed miRNAs and their abundance with comprehensive heat mapping software dedicated to displaying gene expression data. For the TCGA, GEO, and ArrayExpress studies, a two-tailed Student's* t*-test was performed. All statistical tests with a *p*-value of less than 0.05 were considered significant.

## Results

### Screening of candidate miRNAs from public datasets

**Figure 1:**
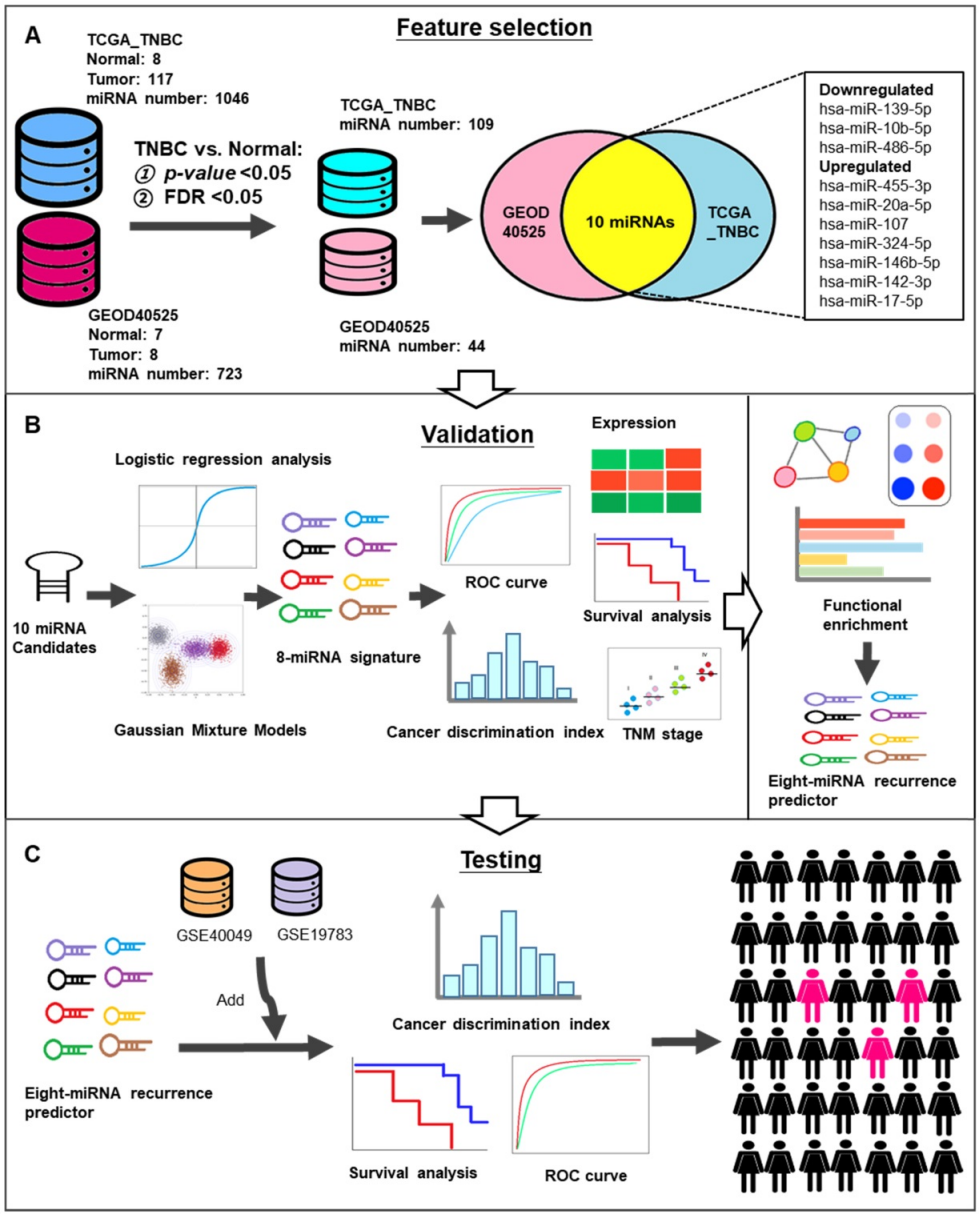
** Schematic workflow for the identification of recurrence-related miRNA predictor(s) in TNBC. (A)** 125 TNBC tissues and 15 adjacent normal tissues were obtained from two different datasets (TCGA_TNBC and GEOD-40525). The 10 candidate miRNAs were intersected from these datasets. **(B)** These 8 miRNAs analyzed by expression level, Kaplan-Meier curves, TNM classification and GSEA for functional validation. **(C)** The GSE40049 and GSE19783 were used to test the predictive accuracy.

To screen significant biomarkers and verify potential candidate miRNAs in TNBC, we incorporated NGS and microarray data. The workflow of the study is shown in Figure 1. A total of 125 TNBC tissues and 15 adjacent normal tissues were obtained from two different datasets (TCGA_TNBC and GEOD-40525). A total of 1046 and 723 miRNAs were expressed in TCGA_TNBC and GEOD-40525, respectively. Next, we set the *p*-value and the FDR threshold as less than 0.05. Then, 109 and 44 miRNAs were reserved in TCGA_TNBC and GEOD-40525, respectively. Finally, 10 candidate miRNAs were common to both the TCGA_TNBC and GEOD-40525 datasets (Figure 1A). The clinicopathological characteristics of the datasets are shown in Table 1. A detailed list of the 10 miRNAs generated by the Venn diagram analysis is provided in Table 2. Furthermore, we used these 10 candidates to verify the miRNA signature by logistic regression and GMM analysis. Afterward, we established an 8-miRNA signature according to the AUC value for tumor relapse (Figure 1B). Since the 8-miRNA signature may be a prognostic biomarker, and an independent study of GSE40049 and GSE19783 was used to validate its predictive accuracy. According to the prediction results, we identified high-risk groups of TNBC patients who require active treatment in order to increase their survival rate (Figure 1C).

**Figure 2:**
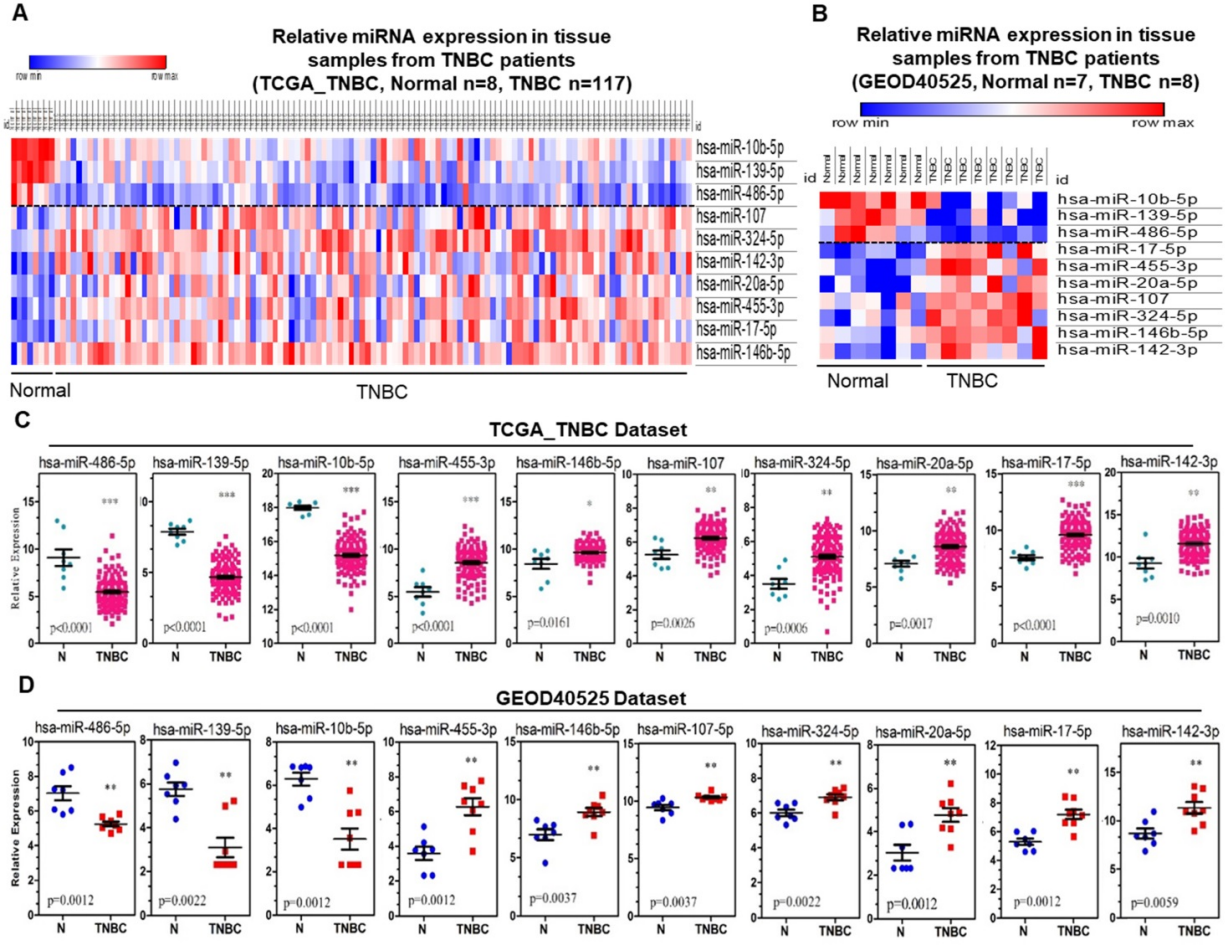
** The ten candidate miRNAs were aberrantly expressed in TNBC samples from the TCGA_TNBC and GEOD-40525 datasets. (A)** Heatmap of miRNA sequencing expression from the TCGA_TNBC dataset. The expression of meta-signature miRNAs between TNBC and noncancer groups (adjacent normal tissue). **(B)** Heatmap of miRNA array expression from the GEOD-40525 dataset. The expression of 10 meta-signature miRNAs between TNBC and noncancer groups (adjacent normal tissue). Adjacent: adjacent normal; TNBC: triple-negative breast cancer. **(C)** The expression of 10 miRNAs between 8 adjacent normal (N) and 117 TNBC tissues from TCGA_TNBC dataset. **(D)** The expression of 10 miRNAs between 7 adjacent normal (N) and 8 TNBC tissues from the GEOD-40525 dataset. The *p-values* were calculated using Student's t-test. *p<0.05;**p<0.01;***p<0.0001.

**Figure 3:**
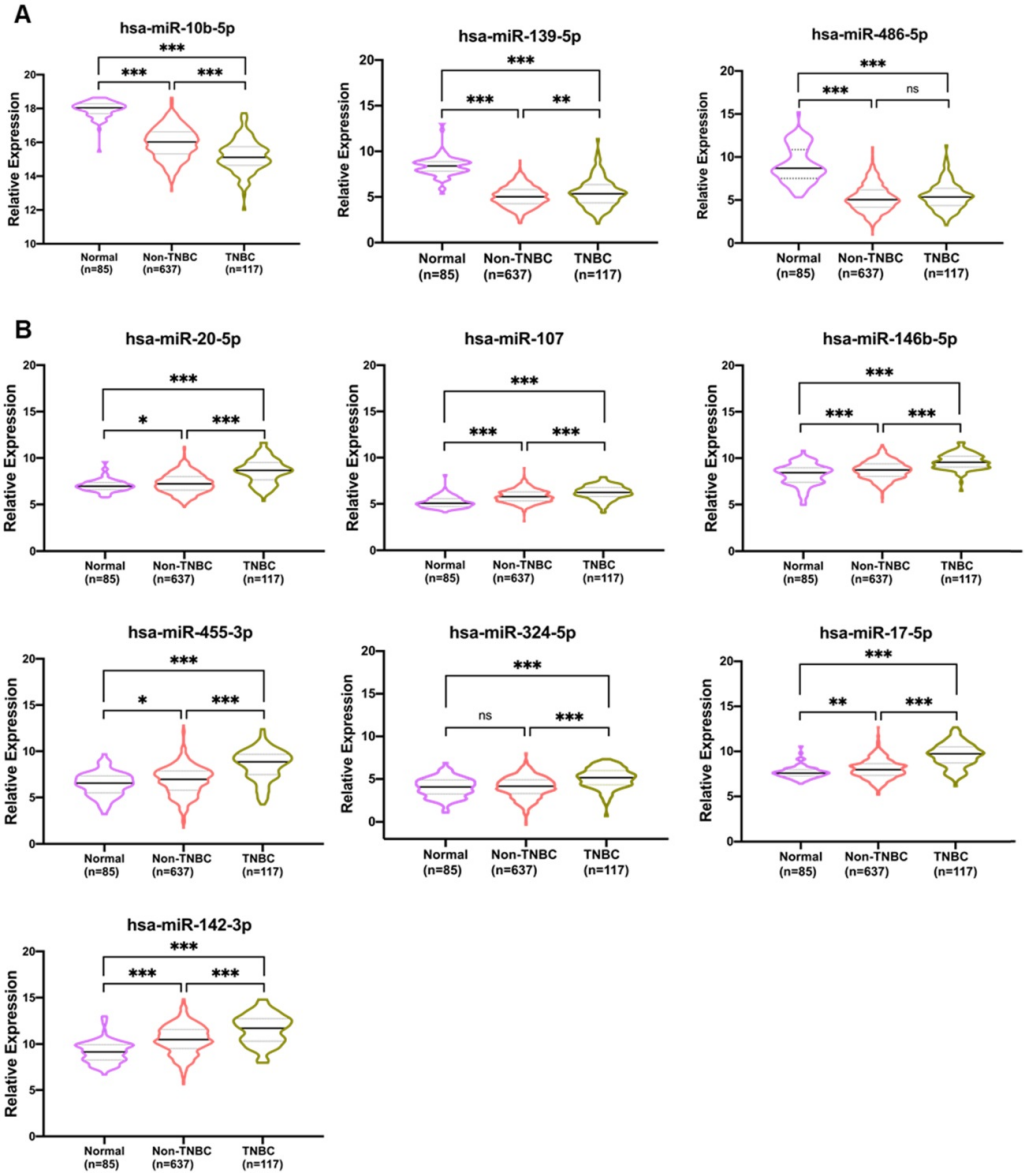
** Comparison of 10 candidate miRNAs in TNBC and non-TNBC.** The bell-shaped curve of ten miRNAs between 85 normal (including 8 and 77 adjacent normal of TNBC and non-TNBC), 117 TNBC and 637 non-TNBC cases from TCGA_TNBC dataset. **(A)** Three downregulated miRNAs in which hsa-miR-486-5p weren't significant between TNBC with non-TNBC patients in 10 candidates. **(B)** Seven upregulated miRNAs were all significant between TNBC with non-TNBC patients in 10 candidates. The *p-value*s were calculated using the Student's t-test. *p<0.05; **p<0.01;***p<0.0001; ns is not significant.

A heatmap was generated representing the expression of the 10 candidate miRNAs distinguished from adjacent normal and tumor tissues for both the TCGA_TNBC and GEOD-40525 datasets combined. The expression levels of hsa-miR-486-5p, hsa-miR-139-5p and hsa-miR-10b-5p were downregulated, and the expression levels of hsa-miR-107, hsa-miR-146b-5p, hsa-miR-142-3p, hsa-miR-17-5p, hsa-miR-455-3p, hsa-miR-324-5p and hsa-miR-20a-5p were upregulated in both the TCGA_TNBC and GEOD-40525 datasets (Figure 2A-B). The comparisons of the expression levels of the 10 candidate miRNAs between the tumor and adjacent normal groups revealed that the differences were statistically significant (all *p*-value <0.05) in the TCGA_TNBC and GEOD-40525 datasets (Figure 2C-D). Based on the above observations, we assessed the specificity and sensitivity of the 10 miRNAs for diagnosis by ROC analysis (Table S1). Additionally, previous studies found that these 10 miRNAs are involved in several cancers (Figure S1). According to the TCGA_TNBC data, expression of the 10 candidates in non-TNBC patients was significant compared to expression in TNBC patients. Only hsa-miR-486-5p of the 3 downregulated miRNAs was not significant (Figure 3A). The hsa-miR-20-5p, hsa-miR-107, hsa-miR-146b-5p, hsa-miR-455-3p, hsa-miR-324-5p, hsa-miR-17-5p, hsa-miR-142-3p were extremely significant in 7 upregulated miRNAs between TNBC with non-TNBC patients (Figure 3B). The results showed that these 10 candidate miRNAs were very different in TNBC and non-TNBC patient samples.

These results suggested that the expression levels of aberrantly expressed miRNAs were consistent among individual studies (TCGA_TNBC and GEOD-40525). Thus, these 10 miRNA candidates might be a promising parameter in patients with TNBC.

### Establishment of the 8-miRNA signature for TNBC recurrence prediction with the training set

**Figure 4:**
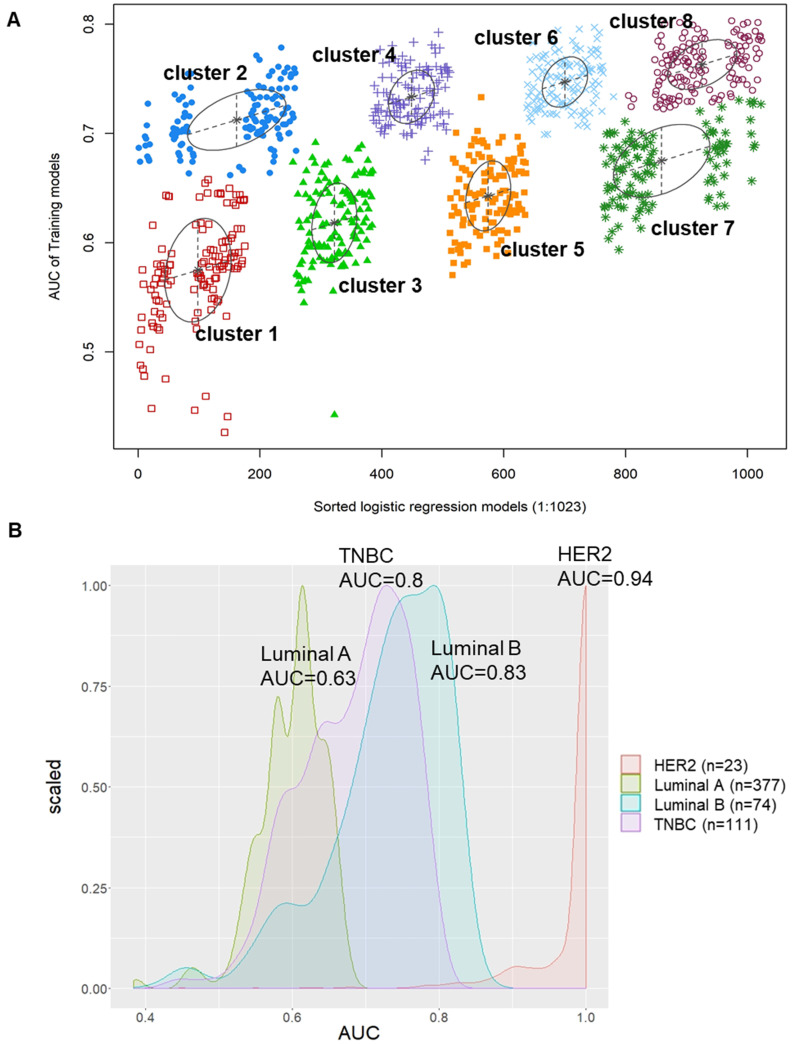
** The pattern of AUC and 1023 logistic regression models were based on Gaussian finite mixture models. (A)** The pattern of the logistic regression model correlated with the AUC scores and was identified by a Gaussian mixture. There are eight clusters of 1023 combinations. **(B)** A total of 1023 combinations correlated with the AUC scores in four BC subtypes.

To implement predictive modeling, we used logistic regression analysis to evaluate the association between the expression values of each of the 10 miRNA candidates as well as the AUC values that were screened in the patient DFS analysis. There were a total of 1023 formulas from the logistic regression model of the 10 miRNA candidates. Furthermore, we used decisive GMM-based clustering, which is a very feasible approach and has a good clustering performance 27-29. Then, we clustered gene sets by the GMM (instead of the 1023 formulas) and AUCs into the eight clusters in our proposed algorithm. Afterward, we selected one of the eight clusters that had the highest AUC as our signature to predict the relapse of TNBC patients. Hence, a miRNA candidate risk score model for recurrence was developed by integrating the expression data of the 8 miRNAs. The hsa-miR-139-5p, hsa-miR-107, hsa-miR-486-5p, hsa-miR-10b-5p, hsa-miR-146b-5p, hsa-miR-455-3p, hsa-miR-20a-5p and hsa-miR-324-5p signatures showed an average accuracy of 0.8031 by the GMM classifier in one of the 1023 formulas, as shown in Figure 4A. Additionally, the accuracy of miRNAs as well as the 8-miRNA and any 7-miRNA signatures to distinguish between recurrent and nonrecurrent patients in the TCGA_TNBC dataset by an ROC test is provided in Table S2. We also used this formula to predict and compare the AUC values of the luminal A (AUC=0.7; green), luminal B (AUC=0.83; blue), HER2 (AUC=0.94; red) and TNBC (AUC=0.8; purple) subtypes, as shown in Figure 4B. The results suggested that the AUC values of these 10 candidates in HER2, luminal B, and TNBC patients were better than those in luminal A patients for predicting relapse. However, the AUC values of 8-miRNA combinations in TNBC were limited to 0.8 and were less than those of the other subtypes.

**Figure 5:**
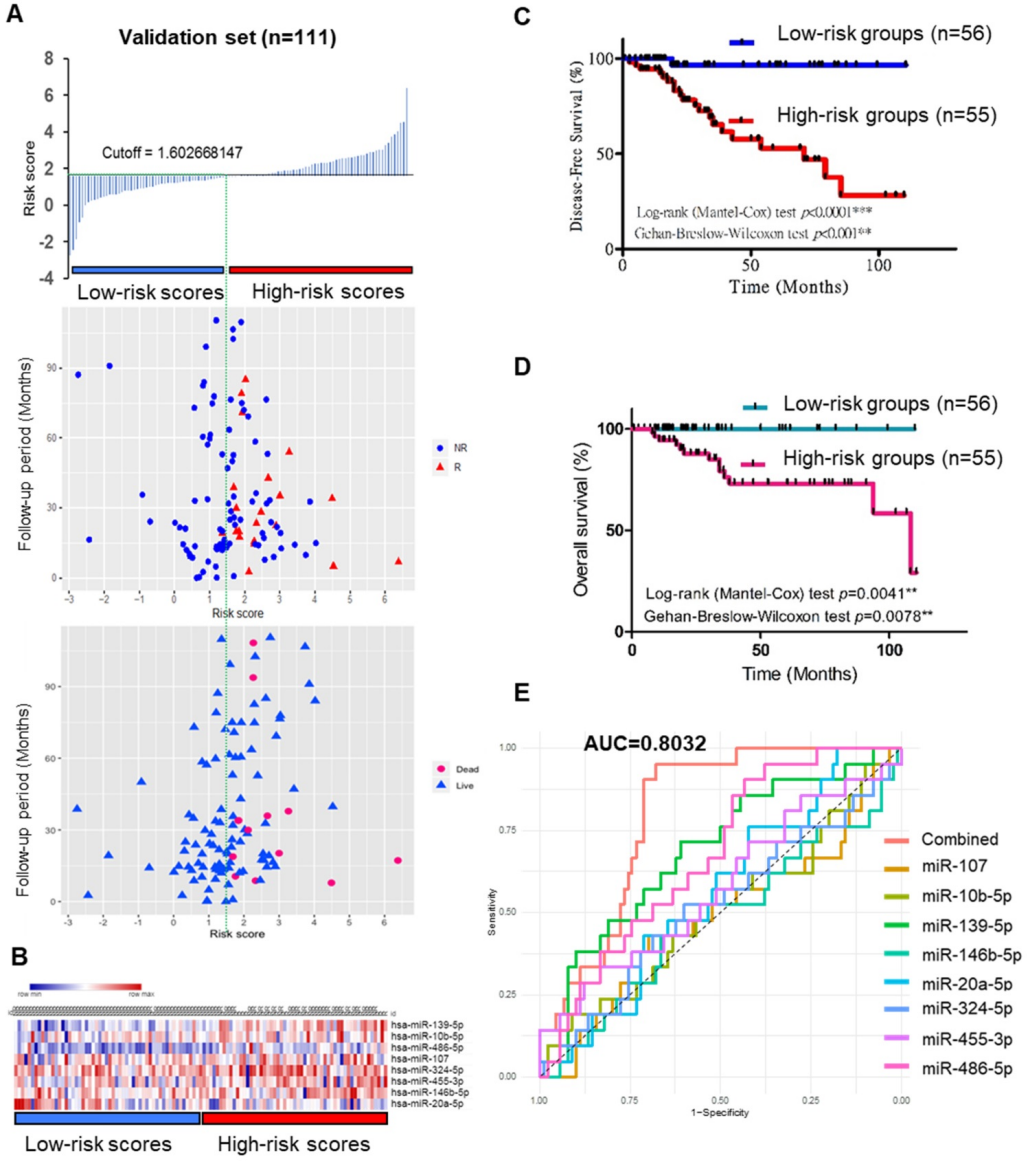
** Predictive value of the 8-miRNA signature in 111 TNBC patients. (A)** The 8-miRNA signature risk score distribution with the DFS and OS status of patients. The colorgram of 8-miRNA expression profiles of high- and low-risk groups with TNBC. The blue line represents the median miRNA signature cutoff dividing patients into low- and high-risk groups. **(B)** The expression of heatmap in 8 miRNAs for 111 TNBC patients. **(C)** Kaplan-Meier estimates of the low- and high-risk groups in DFS for the training set. **(D)** Kaplan-Meier estimates of the low- and high-risk groups in OS for the training set.** (E)** ROC for TNBC recurrence by the miRNA signature between patients with or without recurrence in the combined or respective miRNAs. The 8 combined miRNAs had a stronger predictive value than a single miRNA. OS: overall survival; DFS: disease-free survival; R: recurrence; NR: nonrecurrence.

To validate the prognostic role of this 8-miRNA signature, the miRNA risk score was calculated as follows: the combination miRNA panel= (0.02554× expression value of miR-139) + (-0.000005284× expression value of miR-10b) + (-0.0003305× expression value of miR-486) + (0.008664× expression value of miR-107) + (0.003201× expression value of miR-324) + (0.001031× expression value of miR-455) + (0.000474× expression value of miR-146b) + (-0.001575× expression value of miR-20a). By dividing the risk score according to its median (median=1.602), 111 patients were stratified into high-risk (n=55) and low-risk (n=56) groups (6 patients did not have OS or DFS data in the TCGA_TNBC dataset) (Figure 5A-B). Moreover, Kaplan-Meier survival analysis of the 8-miRNA signature was used to compare the high-risk group with the low-risk group regarding patient DFS and OS. In the analysis, we confirmed that the high-risk group had a significantly higher recurrence and death rate than the low-risk group (Figure 5C-D), and more significantly, the ROC curve further demonstrated that the risk score model was able to effectively predict the recurrence of TNBC patients. Additionally, the AUC value of the 8-miRNA signature was 0.8032 (Figure 5E). The 8-miRNA functional roles of TNBC are shown in Table S3 and are involved in TNBC growth, metastasis, chemoresistance, immunomodulators, relapse, and apoptosis.

These results further support that the combination of the 8-miRNA signature significantly improved the prognostic value. Patients in the high-risk group had a higher relapse and death probability than those in the low-risk group.

### Survival analysis of the prognostic miRNA signature in TNBC

**Figure 6:**
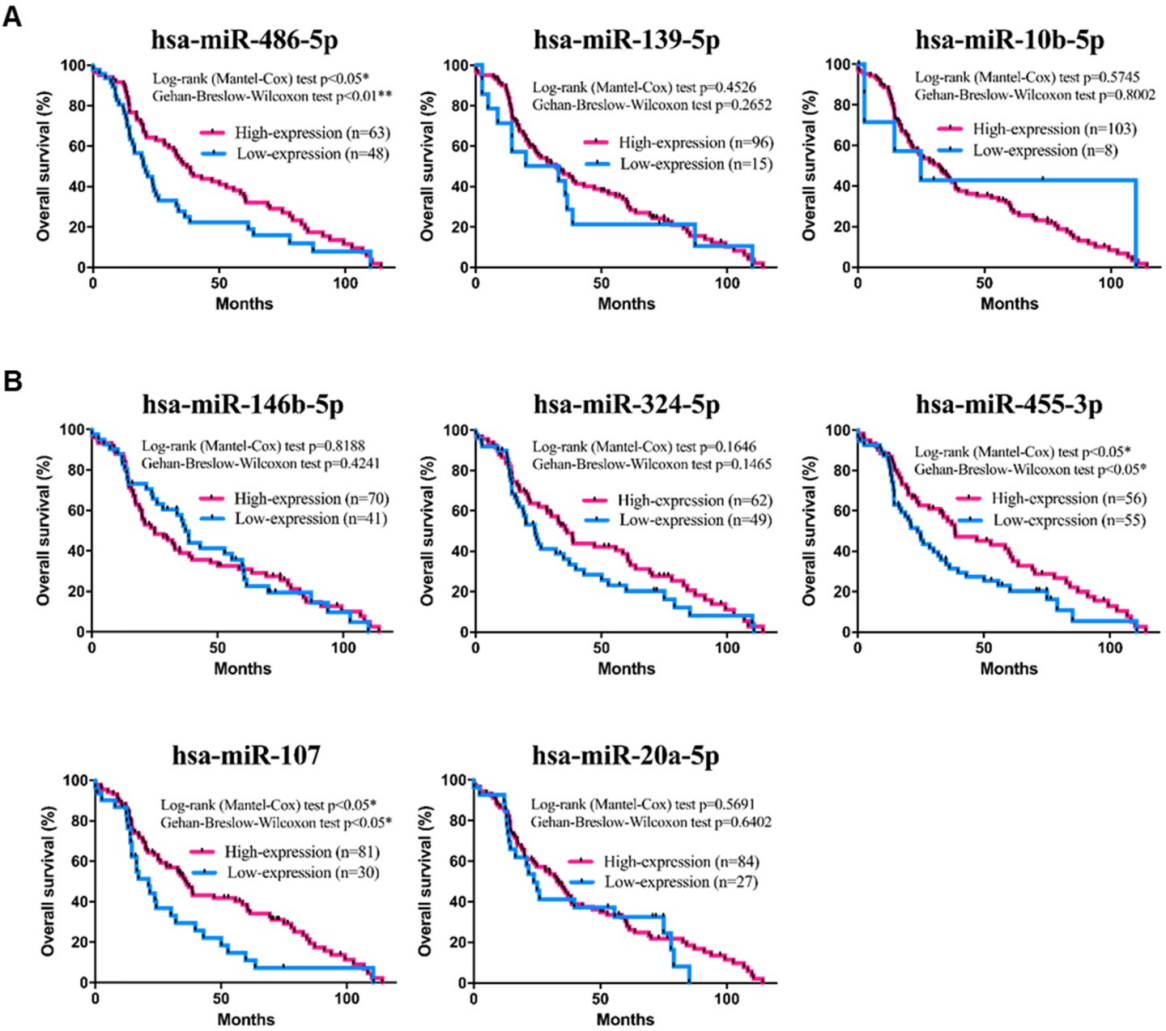
** Kaplan-Meier survival analysis estimates the OS of TNBC patients according to the expression of these 8 miRNAs**. There was a total of 111 patients in the validation set (TCGA_TNBC). **(A)** The three downregulated miRNAs of hsa-miR-486-5p is significant in OS of patients with TNBC. **(B)** The upregulated miRNAs of hsa-miR-455-3p and hsa-miR-107 are significant in OS of patients with TNBC. The *p-value*s were calculated using Log-rank and Gehan-Breslow-Wilcoxon tests. *p<0.05.

**Figure 7:**
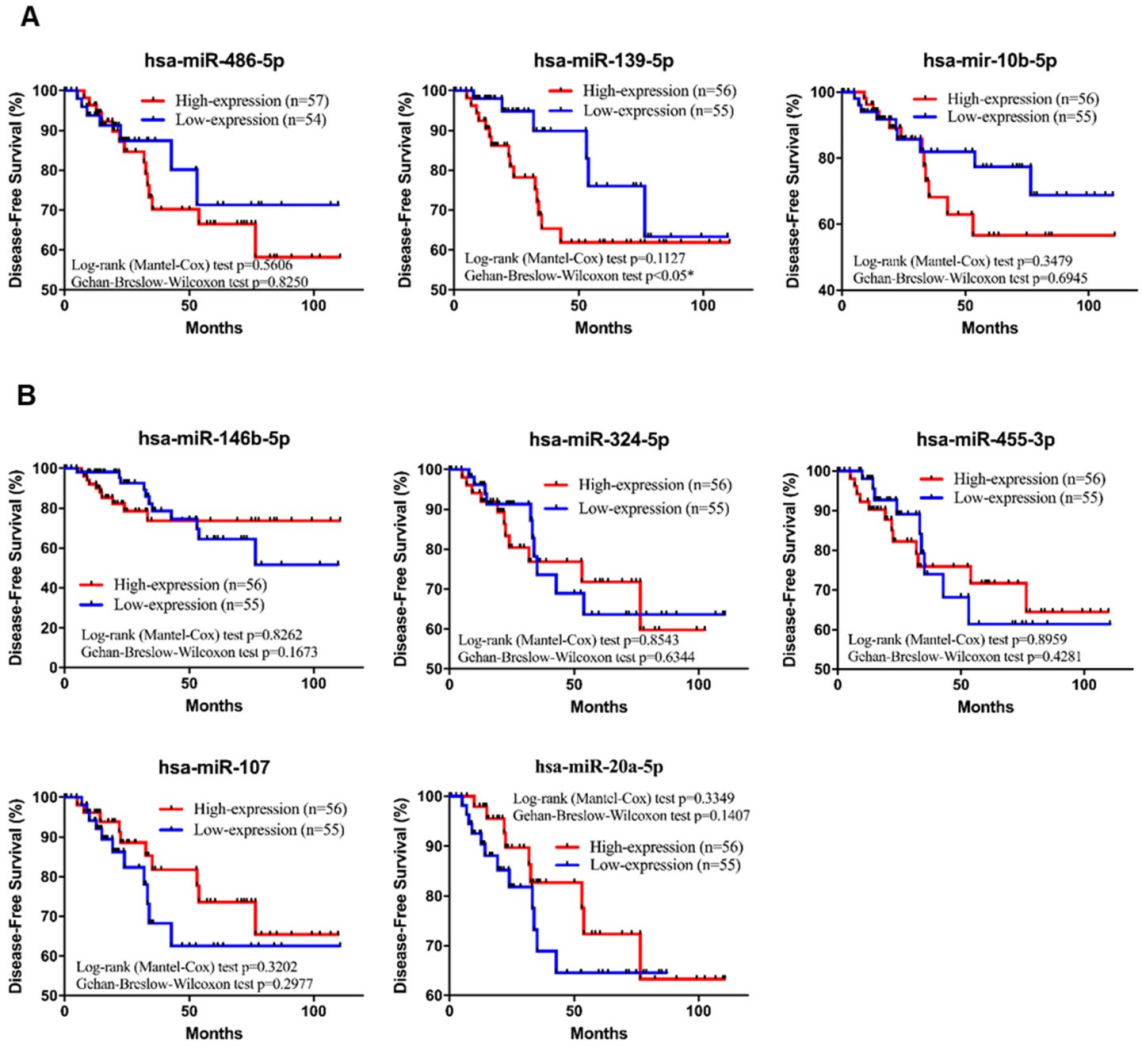
** Kaplan-Meier survival analysis estimates the disease-free survival of TNBC patients according to the expression of these 8 miRNAs**. A total of 111 patients were included in the validation set (TCGA_TNBC). **(A)** The three downregulated miRNAs of hsa-miR-139-5p is significant in DFS of patients with TNBC. **(B)** All of the upregulated miRNAs are not significant in DFS of patients with TNBC. The *p-value*s were calculated using Log-rank and Gehan-Breslow-Wilcoxon tests. *p<0.05.

**Figure 8:**
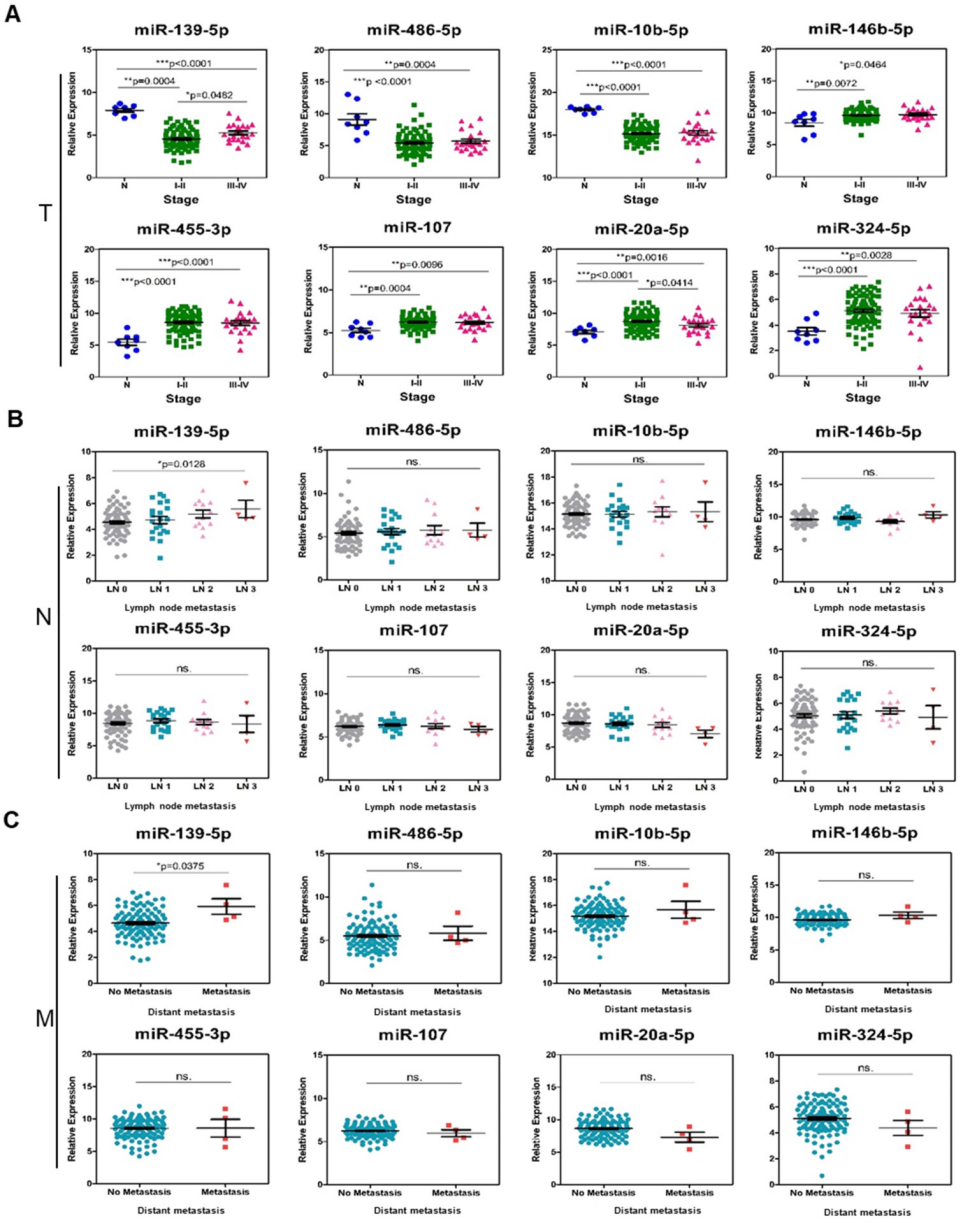
** The difference in 8-signature miRNA expression in subgroups divided by TNM classification. (A)** 111 TNBC patients with 8 N vs. 89 stage I-II vs. 22 stage III-IV. The *p-value*s were calculated with the Kruskal-Wallis test. **(B)** 111 TNBC patients with 74 LN0 vs. 21 LN1 vs. 12 LN2 vs. 4 LN3. The *p-value*s were calculated with the Kruskal-Wallis test. **(C)** 111 TNBC patients with 107 no metastasis vs. 4 metastasis. The *p-value*s were calculated using Student's t-test. *p<0.05; **p<0.01;***p<0.0001; ns is not significant. N: adjacent normal; T: tumor stage; LN: lymph node; M: metastasis.

To further investigate the specific association of the 8 individual miRNAs with clinical characteristics regarding the OS and DFS of TNBC patients, a comprehensive survival analysis was performed with the Kaplan-Meier method. In the analysis, the results suggested that three miRNAs (hsa-miR-455-3p, hsa-miR-107 and hsa-miR-486-5p) were significantly associated with OS (*p-value*<0.05; Figure 6A-B). In addition, the DFS analysis results suggested that hsa-miR-139-5p was significantly associated with DFS (*p-value*<0.05; Figure 7A-B). These results suggested that hsa-miR-139-5p was correlated with recurrence in TNBC patients. Nevertheless, hsa-miR-455-3p, hsa-miR-107 and hsa-miR-486-5p were associated with OS. To investigate the main prognostic factors correlated with the TNM classification for diagnosis, tumor size, lymph node status and distant metastasis were used to represent the main prognostic factors. Only hsa-miR-139-5p was significantly expressed in tumor stages I-II (early stage) and III-IV (*p-value*<0.05; Figure 8A). We also found that hsa-miR-139-5p was highly correlated with lymph node metastasis (LNM; *p-value*<0.05; Figure 8B) and highly expressed in distant metastasis (*p-value*<0.05; Figure 8C). The 8-miRNA signature was assessed in the early stage of TNBC with the distribution of the 8-miRNA signature with risk scores and the recurrence status of the combined 91 patients (stage I and II) from the TCGA_TNBC dataset. Patients with high-risk scores tended to experience increased relapse compared with patients with low-risk scores (AUC=0.8225; Figure S2).

As noted above, these results indicated that hsa-miR-139-5p may play an important role in the progression and metastasis of TNBC. The 8-miRNA signature is a predictor for the recurrence of early stage TNBC in patients.

### Identification of gene sets enriched with the 8-miRNA signature-based risk score

**Figure 9:**
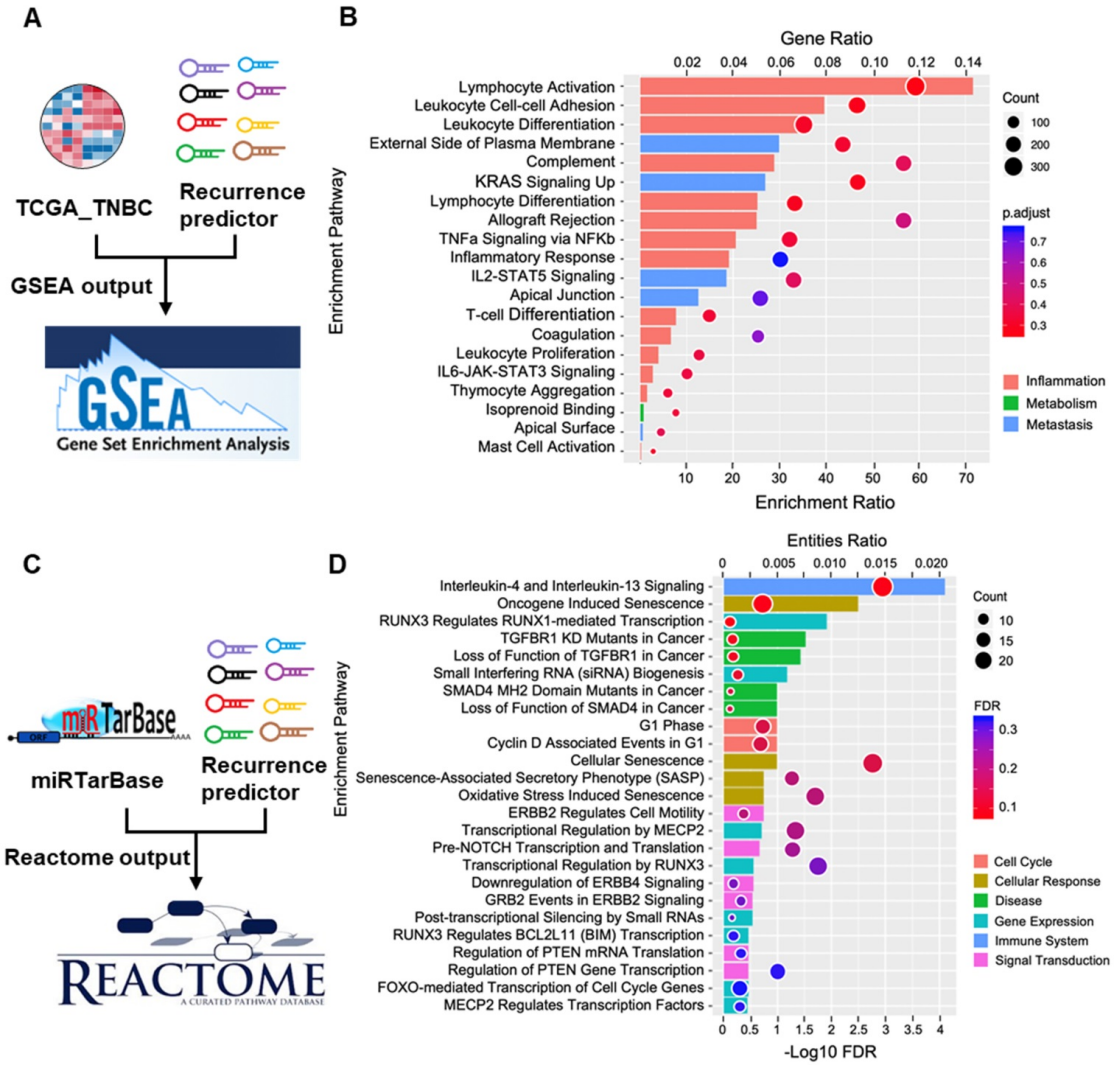
** Network of enrichment analysis for the 8-miRNA recurrence predictor of TNBC. (A)** The workflow showed that the mRNA expression of TCGA_TNBC and the 8-miRNA recurrence predictor were enriched with GSEA. **(B)** The bubble pattern and bar chart shows the top 20 enrichment pathways with GeneRatio, gene count and p.adjust (FDR). The Inflammatory regulation and metastasis correlated with gene enrichment. **(C)** The workflow showed that miRTarBase was combined with the eight-miRNA recurrence predictor and enriched with Reactome. **(D)** The bubble pattern shows the top 25 enrichment pathways with entities.ratio, entities.found (count) and entities.FDR. The bar chart demonstrates that the gene sets involved in the immune system, cellular response, gene expression and disease were significantly enriched in pathways related to the eight-miRNA recurrence predictor.

To comprehensively study the interaction between miRNAs and their functions, GO and Hallmark pathway analyses for the 8-miRNA signature were performed in the high-risk group (Figure 9A). Functional enrichment analysis revealed that the 8-miRNA signature was enriched in inflammation, metastasis and metabolism, and the top 20 pathways are shown in Table S4 and Figure S3. Accordingly, we calculated the enrichment ratio, which is the normalized enrichment score (NES) × GeneRatio (enrichment gene count/total gene count), and then ranked this ratio. The bubble chart shows that the 8-miRNA signature was correlated with TNF-α/NF-κB signaling, thymocyte aggregation, mast cell activation, T cell differentiation, inflammatory responses and cell-cell adhesion. Moreover, the top 10 sets from GSEA with Hallmark gene sets showed that most pathways and genes are critical for inflammatory regulation, and cancer metastasis was associated with a high-risk score. The top 10 GO pathway gene sets were also associated with lymphocyte activation, cell-cell adhesion and the external side of the plasma membrane, which are essential for the inflammatory response and tumor progression (Figure 9B).

To further confirm which biofunctions are correlated with this 8-miRNA signature, we used another approach. The flow chart in Figure 9C combines the miRNA targets from miRTarBase with the 8-miRNA candidates to identify their potential targets. Then, Reactome, which is a functional enrichment tool, was used to align the targets and their biofunctions. Next, a bubble chart was used to show the 8-miRNA signature according to the entities.ratio, entities.found and entities.FDR functions. The results showed that the 8-miRNA signature was correlated with interleukin-4 and interleukin-13 signaling, cellular senescence, transcriptional regulation by RUNX3, transcriptional regulation by MECP2 and oxidative stress-induced senescence. Furthermore, the top 25 functional pathways were ranked with entities. The FDRs are shown in Table S5. The bar chart demonstrates that the resulting pathways are essential for the immune system, cellular response, gene expression, cancer and signal transduction (Figure 9D).

These results suggesed that the 8-miRNA signature is most involved in inflammation and cancer metastasis. This finding might be due to immune escape to promote tumor recurrence, which consequently might have significantly contributed to patients with high-risk scores having higher relapse and death rates than patients with low-risk scores. Therefore, this 8-miRNA signature is defined as the 8-miRNA recurrence predictor of TNBC in this study.

### Validation of the miRNA signature for TNBC recurrence prediction by the validation set

**Figure 10:**
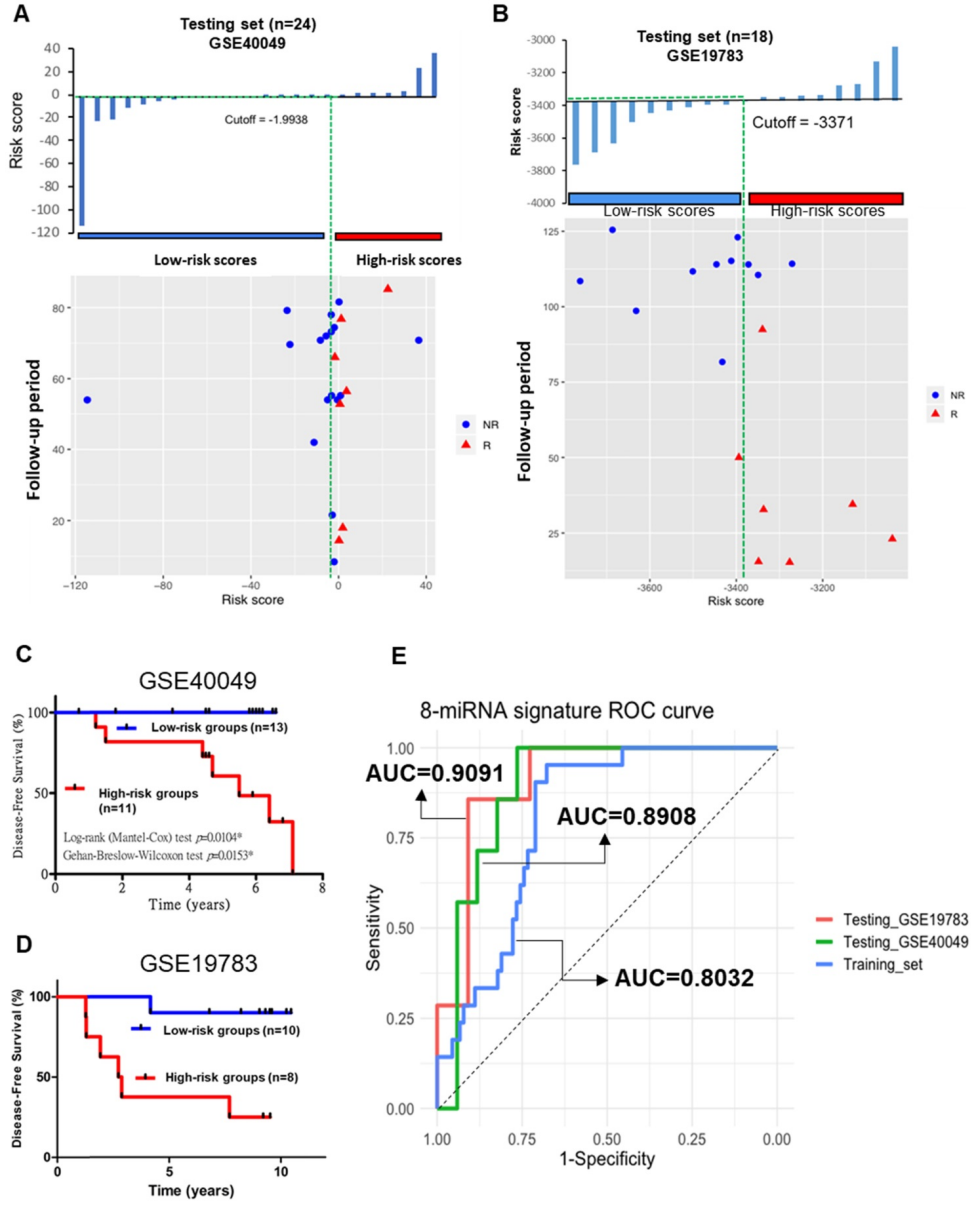
** Predictive value of the 8-miRNA signature for TNBC in the testing study. (A)** The 8-miRNA signature risk score distribution with the DFS status of patients. The colorgram of 8-miRNA expression profiles of high- and low-risk groups with TNBC. The green line represents the median miRNA signature cutoff dividing patients into low- and high-risk groups in GSE40049. **(B)** The 8-miRNA signature risk score distribution with the DFS status of patients. The colorgram of 8-miRNA expression profiles of high- and low-risk groups with TNBC. The green line represents the median miRNA signature cutoff dividing patients into low- and high-risk groups in GSE19783. **(C)** Kaplan-Meier estimates of the low- and high-risk groups in DFS for the testing set GSE40049. **(D)** Kaplan-Meier estimates of the low- and high-risk groups in DFS for the testing set GSE19783. **(E)** ROC curve for TNBC patient relapse by the 8-miRNA signature with/without recurrence in the combined or respective miRNAs. The AUC supports that the 8-miRNA signature best predicts in both the training (TCGA_TNBC) and testing sets (GSE40049 and GSE19783). R: recurrence; NR: nonrecurrent.

To validate the prognostic role of this 8-miRNA signature, we applied the same miRNA signature obtained from testing to an additional 60 TNBC patients in independent cohorts. The expression in the validation cohort GSE40049, GSE19783, and E-MTAB-1989 datasets was assessed and comprised of recurrence events and no recurrence events (the clinicopathological characteristics are shown in Table S6). We performed logistic regression analysis using the same 8-miRNA signature to diagnose and predict the probability of patient recurrence. According to the median risk score (median=-1.9938), 24 patients were stratified into high-risk (n=11) and low-risk (n=13) groups in GSE40049 (Figure 10A). In addition, according to the median risk score (median=-3371), 18 patients were stratified into high-risk (n=8) and low-risk (n=10) groups in GSE19783 (Figure 10B). Kaplan-Meier survival analysis with the 8-miRNA signature was used to compare the DFS of patients in the high-risk and low-risk groups. In the analysis, we confirmed that the 8-miRNA signature in the high-risk group was associated with a significantly higher recurrence in patients from the GSE40049 (Figure 10C), GSE19783 (Figure 10D), and E-MTAB-1989 datasets (data not shown). We analyzed the AUC values between the training and validation sets, which were 0.8961 (GSE19783) and 0.9062 (GSE40049) in the validation sets compared to 0.8032 in the training set (Figure 10E). Hence, the ROC curve showed that the 8-miRNA signature in the validation sets was better than that in the training set.

In summary, the combination of the 8-miRNA signature in the validation sets showed a significantly improved prognostic value (AUC=0.8961 and 0.9062). Patients in the high-risk groups had more recurrence and death than those in the low-risk groups.

## Discussion

In this study, we identified a total of 8 miRNAs as a signature that is associated with tumor recurrence in TNBC patients from the training sets, TCGA_TNBC and GEOD-40525. We further verified that these findings were consistent in three validation sets, GSE40049, GSE19783 and E-MTAB-1989. The prognostic risk score of recurrence in TNBC patients and individual current prognosis regimens based on precise predictions are important. Our results showed that patients with high-risk scores according to this 8-miRNA signature have increased cancer relapse and decreased survival. In addition, previous studies have reported that these miRNAs are correlated with several cancer types, including colorectal cancer, BC, lung cancer, gastric cancer, prostate cancer, endometrial cancer, pancreatic cancer, etc. These tumor-associated miRNAs may play a crucial role in the pathogenesis, tumor progression and prognosis of TNBC 30-34.

The World Health Organization (WHO) successfully separates BC into TNBC and non-TNBC according to the histopathological characteristics 35. We explored the expression levels of 10 miRNAs in TNBC and non-TNBC and compared them to corresponding levels in adjacent normal tissues. First, the 10 miRNAs were significantly expressed between the two analyzed TNBC and non-TNBC groups. Second, the expression levels were very different between the TNBC and non-TNBC groups for miR-139-5p, miR-107, miR-10b-5p, miR-146b-5p, miR-17-5p, miR-142-3p, miR-455-3p, miR-20a-5p and miR-324-5p but not for miR-486-5p (*p-value* of 0.2137). Furthermore, based on our findings, an 8-miRNA signature given by the miR-139-5p, miR-107, miR-486-5p, miR-10b-5p, miR-146b-5p, miR-455-3p, miR-20a-5p and miR-324-5p expression levels was demonstrated to significantly influence the prognosis of TNBC patients but not non-TNBC patients.

In this study, the 8 miRNAs can predict the relapse of TNBC in the combination of logistic regression. For individuals, each miRNA also regulates the progression of TNBC in previous experimental studies by up- or downregulation of expression levels. Among them, 5 miRNAs upregulated in TNBC improve the metastasis progression of TNBC (such as miR-107, miR-20a-5p, and miR-455-3p) 36-38, proliferation (such as miR146b-5p and miR-455-3p) 32, 38, and apoptosis (such as miR-20a-5p and miR-324-5p) 39, 40. The downregulated miRNAs were miR-139-5p, miR-10b-5p, and miR486-5p, which are involved in chemoresistance and metastasis 31, 41-46.

These miRNAs are involved in the complex regulation of TNBC progression, and most of them are associated with metastasis and resistance. Even though all of them are related to TNBC development, it is still difficult to determine the fate of cancer development based on each miRNA. Due to the complexity of the genetic network, tumor progression is more likely to depend on a group of critical miRNAs rather than a single one. Therefore, the prognosis analysis might not always be consistent with the unique miRNA expression level (Figs 6, 7). The evident reason is that miRNAs play a pleiotropic role in cancer. Some studies have indicated the pleiotropic role of miRNAs in various cancers, such as miR-107 and miR-146-5p 32, 47. For TNBC, miR-107 regulates tumor progression by both oncogenic and suppressor effects on metastasis. These studies implied that relapse prediction might depend on a group of critical miRNAs, and this hypothesis was verified by the significant association of OS analyses in our study (Figs 5, 10).

Previous studies did not investigate these 8 miRNAs as a signature to predict the relapse of TNBC patients. In addition, the 8-miRNA signature was analyzed for DFS and OS. The findings suggest that only miR-107, miR-146b-5p, miR-455-3p, miR-486-5p and miR-139-5p have statistical significance in TNBC patients. However, we used the 5-miRNA signature to predict the recurrence of patients, and it had poor prognostic results, with an AUC of 0.673 (Figure S4). Similarly, we also tried to calculate a 7-miRNA signature to predict the recurrence of TNBC patients. The data showed that the 7-miRNA signature (AUC of 0.8032) has very similar accuracy to the 8-miRNA signature (AUC of 0.8005) (Figure S5). Most of the genes are targeted by more than one miRNA, and these miRNAs may target the same or different genes in similar functional pathways 48, 49.

These reasons lead to differences in the predictions according to the 5- or 8-miRNA signature based on RNA-RNA crosstalk and ceRNA-ceRNA regulation. Juan Xu et al. provided constructive suggestions regarding miRNA-miRNA crosstalk. They consider miRNA crosstalk based on genomic similarity, regulatory networks, functions and phenomics 50. In addition, a growing number of studies have tried to investigate ceRNA-ceRNA regulation in specific cancer types. The competing endogenous RNAs (ceRNA) hypothesis assumes that the RNA transcript that covers miRNA recognition elements (MREs) can sequester miRNAs from other targets sharing the same MREs, thereby regulating their expression 51. Hence, the combined signature is crucial for cancer risk prediction since it integrates the multifactorial nature of cancer and tumorigenesis, which is imperative for the personalization of patient care.

Libero Santarpia et al. demonstrated that a four-miRNA signature (miR-18b, miR-103, miR-107, and miR652) may assist in accurately predicting tumor relapse and OS in patients with TNBC 52. We performed a ROC analysis by this four-miRNA signature and compared it with our 8-miRNA signature. The Figure S6 suggested that our 8-miRNA signature predicted DFS ability better than the 4-miRNA signature in TCGA_TNBC and combined GSE19783 and E-MTAB-1989 data. Additionally, Kaplan-Meier analysis of miR-18b, miR-103, miR-107, and miR652 expression is shown in Figure S7. OS in TNBC patients with miR-107 expression levels of survival was significant. However, no miRNAs in the DFS of TNBC patients were significant (Figure S8). The Figure S9 shows the expression of the four miRNAs in the TNM classification.

miR-139-5p was highly correlated with TNM stage and was able to distinguish between different stages (I-II vs. III-IV stage, *p*<0.05), nodes (LN0, LN1, LN2 and LN3, *p*<0.05), and metastasis (no metastasis vs. metastasis, *p*<0.05). Several lines of evidence suggest that miR-139-5p is a prognostic biomarker for different cancer types. For example, the EZH2/miR-139-5p axis impeded EMT and LNM in pancreatic cancer 53. MiR-139-5p downregulated VEGFR to inhibit signaling pathways in the development of esophageal cancer 54. MiR-139 could act as an anti-oncomir to suppress primary malignant brain tumor progression by targeting insulin-like growth factor 1 receptor (IGF-1R), associate of Myc 1 (AMY-1) and peroxisome proliferator-activated receptor γ coactivator 1β (PGC-1β), thus inhibiting the PI3K/AKT and c-Myc signaling pathways 55. The tumor suppressor function of miR-139-5p involves targeting HOXA10 to inhibit endometrial cancer cell growth and migration 56. MiR-139-5p was able to regulate the cell motility and invasion of aggressive BC through the TGFβ, Wnt, Rho, and MAPK/PI3K signaling cascades 41. MiR-139-5p directly binds to Rho-associated coiled-coil-containing protein kinase 2 (ROCK2) to suppress cell proliferation and invasion in ovarian cancer (OC) 57. Many studies have identified that the miR-139-5p expression level could serve as a diagnostic, prognostic and therapeutic marker in the future. In addition, low miR-139-5p expression was correlated with poor prognosis in hepatocellular carcinoma (HCC) and glioblastoma multiforme (GBM). However, further research and studies with larger samples are still needed to elucidate its functions 58, 59.

MiRNAs not only play a pivotal role in tumor differentiation but also contribute to biological processes in TNBC. Functional enrichment of the 8-miRNA signature was analyzed with Hallmark and GO annotations. The combined results showed that these miRNAs were highly correlated with inflammatory regulation, tumor metastasis, and metabolism. Many reports confirm that TNBC exhibits the strongest immunogenicity and may provide an option for immunotherapy. For example, CD4+ helper T cells have an immune response pathway via Th1 and Th2 in ER-negative BC. Type I immune responses, such as CD4+ T cells, secrete cytokines (TNF-α, IFN-Υ, CD8+, and IL-2 cytotoxic T cells) to support the destruction of the tissue environment. Moreover, tumor-associated macrophages (TAMs) are composed of M1 and M2 phenotypes and are correlated with macrophage polarization, cytokine profiles and migratory functions 60. Hartman et al. demonstrated that an effective treatment strategy involved suppressing both IL-6 and IL-8 in TNBC. Hence, recent evidence has suggested that activated immune response genes are associated with good prognosis 61, 62. Furthermore, a recent clinical trial used pembrolizumab, which is a high-affinity anti-PD-L1 antibody, in metastatic TNBC patients who express PD-L1. PD-L1 can bind to and activate cytotoxic T cells to prevent T-cell activation and proliferation as well as the release of IL-2. PD-L1 is an important regulatory checkpoint since it prevents excessive adaptive immune responses 63-65. Metastasis in BC is characterized by a distinctive spread to the lungs, liver, brain, and bones via regional lymph nodes. Increasing evidence shows that miRNAs are involved in a variety of processes contributing to tumorigenesis and metastasis in TNBC 66. In recent studies of metastatic BC, miR-10b, miR-20a, miR-139-5p, and miR-486-5p were highly expressed in lymph node metastases 67, 68. In addition, MUC1, which is a cell wall-based mucin glycoprotein present on the apical surface of epithelial cells, is highly expressed in many adenocarcinomas. Pillai K et al. demonstrated that MUC1 overexpression is associated with angiogenesis and chemoresistance in cancer 69-72.

A major question that must be asked is why do miR-107, miR-486-5p and miR-455-3p have statistical significance in OS but not in DFS? Likewise, miR-146b-5p and miR-139-5p were significant in DFS. Zhiying Luo et al. demonstrated that the expression level of miR-107 has also been associated with both DFS and OS. Overexpression of miR-107 and miR-146b-5p is significantly associated with an improved objective response to chemotherapy and promotes cell growth, invasion and glycolysis in colorectal cancer (CRC) 72, 73. In addition, the oncomiR miR-455-3p provides a potential therapeutic target to achieve better clinical outcomes in cancer 74. Both miR-486 and miR-139-5p can be used as biomarkers for cancer recurrence. Although these previous studies were not all focused on TNBC, these 5 miRNAs were all associated with clinical outcomes. Another question to raise is why the expression levels (adjacent normal vs. tumor) of 3 of the 8 miRNAs in GSE40049 were not consistent with the training results (Figure S10). We believe the differences lie in the composition due to the diversity of TNBC tissue. In the training set, the results of the 8-miRNA signature analyses were also used to predict patient relapse. In addition, the platforms applied during training and validation were different. For example, the NGS platforms applied for the quantification of miRNA expression included Illumina HiSeq 2000 miRNA sequencing and Applied Biosystems SOLiD sequencing. Each method has its strengths and weaknesses. In addition, recent studies have discussed that “scientists rise up against statistical significance”. Because of human nature and cognition, different researchers think that a *p-value* of more or less than 0.05 in results can be either statistically significant or statistically insignificant. These common practices suggest that the thresholds of statistical significance can be misleading 75.

Overall, the evidence indicates that this 8-miRNA signature can accurately predict the relapse of TNBC patients and that it will be useful for further clinical prognosis.

## Conclusions

In conclusion, we demonstrated that it is possible to accurately identify clinical outcomes in TNBC patients using an 8-miRNA signature. The 8-miRNA signature could be useful in TNBC according to risk in trials on the adjuvant treatment of patients. Further validation studies in large independent patient cohorts are needed to assess the true clinical value of our findings for TNBC diagnosis and prognosis.

## Supplementary Material

Supplementary figures and tables.

## Websites

- [Internet] TCGA: Human Cancer Genome Project 2017. https://cancergenome.nih.gov/
- [Internet] GEO: Functional genomics data. https://www.ncbi.nlm.nih.gov/geo/
- [Internet] ArrayExpress database: ArrayExpress—a public database of microarray experiments and gene expression profiles. Received September 20, 2006; Revised October 27, 2006; Accepted October 30, 2006. http://www.ebi.ac.uk/arrayexpress
- [Internet] miRTarBase update 2020: a resource for experimentally validated microRNA-target interactions. http://miRTarBase.cuhk.edu.cn/
- [Internet] GSEA: Gene set enrichment analysis: A knowledge-based approach for interpreting genome-wide expression profiles. Eric S. Lander, August 2, 2005. http://software.broadinstitute.org/gsea/index.jsp
- [Internet] Reactome knowledgebase: Reactome: a database of reactions, pathways and biological processes. Received September 15, 2010; Accepted October 10, 2010. https://reactome.org
- [Internet] Venny 2.1: Oliveros, J.C. (2007-2015) Venny. An interactive tool for comparing lists with Venn's diagrams. https://bioinfogp.cnb.csic.es/tools/venny/index.html
- [Internet] GENE-E: Joshua Gould. http://www.broadinstitute.org/cancer/software/GENE-E/

## Figures and Tables

**Table 1:** Clinicopathological characteristics of TNBC patients in this study.

Data set	TCGA_TNBC	GEOD 40525 data set
**Number**		
TNBC	117	8
Adjacent normal	8	7
Total	125	15
**Age (years)**	57.42±14.56	NA
**Preservation type**	Fresh tissue	Fresh tissue
**TNM Stage**		
I	21	NA
II	68	NA
III	21	NA
IV	1	NA
Other	6	NA
**Lymph node metastasis**		
Present	74	5
Absent	37	3
Other	6	0
**Distant metastasis**		
Present	4	NA
Absent	107	NA
Other	6	NA
**Number of deaths**	96	NA
**Median survival (months)**	25.4	NA
**Follow-up period (days)**		
Median	858	NA
Range	1-3472	NA
**Platform**	Illumina HiSeq 2000 miRNA Sequencing, Illumina Genome Analyzer miRNA Sequencing	Agilent 019118 Human miRNA Microarray 2.0

NA: not available. Mean ± standard deviation (SD) were presented.

**Table 2:** The expression of ten candidate miRNAs in TNBC tissue between the TCGA_TNBC and GEOD-40525 datasets.

Data sets		TCGA_TNBC			GEOD_40525		
microRNA	Chromosome	Log2 fold change	p-value	FDR	Log2 fold change	p-value	FDR
**Downregulated**							
hsa-miR-139-5p	11q13.4	-2.895735451	8.33E-33	9.08E-31	-2.662765892	3.76E-04	1.60E-02
hsa-miR-10b-5p	2q31.1	-2.511149588	2.59E-32	1.41E-30	-2.779048673	4.60E-04	1.58E-02
hsa-miR-486-5p	8p11.21	-4.248281522	3.08E-09	8.39E-08	-1.778231191	7.94E-04	1.91E-02
**Upregulated**							
hsa-miR-455-3p	9q32	3.309861939	3.19E-02	3.91E-02	2.688322067	9.74E-04	2.13E-02
hsa-miR-20a-5p	13q31.3	1.950986771	4.29E-02	4.62E-02	1.738424993	3.08E-03	4.36E-02
hsa-miR-107	10q23.31	1.000952191	6.88E-03	1.79E-02	0.883928962	3.40E-03	4.38E-02
hsa-miR-324-5p	17p13.1	1.785810283	7.72E-03	1.87E-02	0.885061916	4.45E-03	5.26E-02
hsa-miR-146b-5p	10q24.32	0.94811004	3.38E-02	3.92E-02	1.951810829	4.45E-03	5.26E-02
hsa-miR-142-3p	17q22	1.986602322	4.99E-02	4.99E-02	2.661047195	5.23E-03	5.00E-02
hsa-miR-17-5p	13q31.3	2.453964607	2.03E-02	3.07E-02	1.900494005	4.49E-04	1.71E-02

FDR: False-discovery rate

## Abbreviations

BC: breast cancer
TNBC: triple-negative breast cancer
Non-TNBC: nontriple-negative breast cancer
ER: estrogen receptor
PR: progesterone receptor
HER2: human epidermal growth factor receptor 2
MDR: multidrug resistance
DFS: disease-free survival
OS: overall survival
miRNA: microRNA
EMT: epithelial-to-mesenchymal transition
CSC: cancer stem cell-like
NGS: next-generation sequencing
TNM: tumor-node-metastasis
TCGA: The Cancer Genome Atlas
GEO: Gene Expression Omnibus
RPM: reads per million
GMM: Gaussian mixture model
GSEA: gene set enrichment analysis
ROC: receiver operating characteristic
AUC: area under the curve
GO: Gene Ontology
WHO: World Health Organization
LN: lymph node
CeRNA: competing endogenous RNAs
R: recurrence
NR: nonrecurrent
