# Supplementary material for: A panel of eight microRNAs is a good predictive parameter for triple-negative breast cancer relapse
Source: Theranostics. 2020 Jul 9;10(19):8771–89. doi: 10.7150/thno.46142 (PMC7392022; doi:10.7150/thno.46142)
Supplement: Supplementary file 1 — Supplementary figures and tables. [file thnov10p8771s1.pdf]

Supporting Information

Title: A panel of eight microRNAs is a good predictive parameter for triple-negative breast cancer relapse

Hsiao-Chin Hong[1,2*], Cheng-Hsun Chuang[3,5*], Wei-Chih Huang[4,5,6], Shun-Long Weng[7,8,9], Chia-Hung Chen[10], Kuang-Hsin Chang[4,5], Kuang-Wen Liao[3,5,11,12✉], Hsien-Da Huang[1,2,5✉]

1. Warshel Institute for Computational Biology, The Chinese University of Hong Kong, Shenzhen, Guangdong Province 518172, China
2. School of Life and Health Sciences, The Chinese University of Hong Kong, Shenzhen, Guangdong Province 518172, China
3. Institute of Molecular Medicine and Bioengineering, National Chiao Tung University, Hsinchu City 30068, Taiwan, ROC
4. Institute of Bioinformatics and Systems Biology, National Chiao Tung University, Hsinchu City 30068, Taiwan, ROC
5. Department of Biological Science and Technology, National Chiao Tung University, Hsinchu City 30068, Taiwan, ROC
6. Come True Biomedical Inc., Taichung 408, Taiwan, ROC
7. Department of Obstetrics and Gynecology, Hsinchu MacKay Memorial Hospital, Hsinchu City 300, Taiwan, ROC
8. Department of Medicine, MacKay Medical College, New Taipei City 252, Taiwan, ROC
9. MacKay Junior College of Medicine, Nursing and Management College, Taipei City 112, Taiwan, ROC
10. Department of Medical Research, Hsinchu Mackay Memorial Hospital, Hsinchu City 30071, Taiwan, ROC
11. Center for Intelligent Drug Systems and Smart Bio-Devices, National Chiao Tung University, Hsinchu City 30068, Taiwan, ROC
12. Graduate Institute of Medicine, College of Medicine, Kaohsiung Medical University, Kaohsiung 80708, Taiwan, ROC

*These authors contributed equally to this work.

☒ Corresponding authors: **Kuang-Wen Liao, Ph.D.,** National Chiao Tung University, 75 Bo-Ai Street, Hsinchu, 300, Taiwan; Email: liaonms@mail.nctu.edu.tw; TEL: +886-3-5729287 ext. 56955. **Hsien-Da Huang, Ph.D.**, The Chinese University of Hong Kong, Shenzhen, H.L. Tu Building, 2001 Longxiang Blvd., Longgang District, Shenzhen, China 518172. Email: huanghsienda@cuhk.edu.cn; TEL: +86 (0) 755 2351 9601.

Table S1. Area under the ROC curve for individual miRNAs from the TCGA_TNBC and GEOD-40525 datasets between adjacent normal and TNBC tissue samples.

**Model set**	**TCGA_TNBC**	***p*-value**	**GEOD-40525**	***p*-value**
**miR-139-5p**	0.9959	<0.0001	0.9464	0.003830
**miR-10b-5p**	0.9968	<0.0001	0.9643	0.002635
**miR-486-5p**	0.9217	<0.0001	0.9821	0.001790
**miR-455-3p**	0.9145	<0.0001	0.9107	0.007799
**miR-324-5p**	0.8665	0.0005449	0.9464	0.003830
**miR-142-3p**	0.8504	0.0009443	0.9107	0.007799
**miR-146b-5p**	0.7553	0.01595	0.9286	0.005499
**miR-107**	0.8194	0.002574	0.9286	0.005499
**miR-20a-5p**	0.8323	0.001682	0.875	0.01512
**miR-17-5p**	0.9135	<0.0001	0.9643	0.002635

Table S2. Area under the ROC curve for seven and eight miRNAs from three public datasets between patients with TNBC recurrence and no recurrence.

TCGA_TNBC in training set			
**Model set**	**Number**	**miRNA combinations**	**AUC**
Model_1	7	RNR~miR-139+miR-10b+miR-486+miR-107+miR-324+miR-455+miR-146b	0.7560847
Model_2	7	RNR~miR-139+miR-10b+miR-486+miR-107+miR-324+miR-455+miR-20a	0.7846561
Model_3	7	RNR~miR-139+miR-10b+miR-486+miR-107+miR-324+miR-146b+miR-20a	0.7481481
Model_4	7	RNR~miR-139+miR-10b+miR-486+miR-107+miR-455+miR-146b+miR-20a	0.8005291
Model_5	7	RNR~miR-139+miR-10b+miR-486+miR-324+miR-455+miR-146b+miR-20a	0.7724868
Model_6	7	RNR~miR-139+miR-10b+miR-107+miR-324+miR-455+miR-146b+miR-20a	0.8015873
Model_7	7	RNR~miR-139+miR-486+miR-107+miR-324+miR-455+miR-146b+miR-20a	0.7994709
Model_8	7	RNR~miR-10b+miR-486+miR-107+miR-324+miR-455+miR-146b+miR-20a	0.7402116
Model_9	8	RNR~miR-139+miR-10b+miR-486+miR-107+miR-324+miR-455+miR-146b+miR-20a	0.8031746

Table S3. The annotation of 8 miRNAs in TNBC by previous experimental studies (UP: Upregulation; DOWN: Downregulation).

Name	Functional role in TNBC	Expression level	Suppl. Ref.
**miR-139-5p**	metastasis	DOWN	[1, 2]
**miR-10b-5p**	chemoresistance, metastasis	DOWN	[3-6]
**miR-486-5p**	immunomodulatory tumor suppressor	DOWN	[7-9]
**miR-107**	metastasis, correlated with relapse	UP/DOWN	[6, 10-13]
**miR-455-3p**	migration, invasion	UP	[14]
**miR-146b-5p**	proliferation, homologous recombination	UP	[15]
**miR-20a-5p**	migration, invasion, growth	UP	[16, 17]
**miR-324-5p**	anti-apoptosis potential, associated with decreased OS	UP	[6, 18]

Suppl. Ref.: Supplementary reference.

Table S4. Gene Ontology and Hallmark pathways correlated with high-risk groups of TNBC patients.

Function	Enrichment pathway	Count	NES	p.adjust	*P* value	GeneRatio	Enrichment ratio
Inflammation	LYMPHOCYTE_ACTIVATION	303	1.93379	0.25821	0.004081633	0.12228	71.64629866
Inflammation	LEUKOCYTE_CELL_CELL_ADHESION	221	1.936	0.2802	0.004040404	0.09258	39.61300356
Inflammation	LEUKOCYTE_DIFFERENTIATION	251	1.93784	0.31003	0.00203252	0.07125	34.65389229
Metastasis	EXTERNAL_SIDE_OF_PLASMA_MEMBRANE	183	1.95803	0.32262	0.001937985	0.08345	29.90084072
Inflammation	COMPLEMENT	182	1.48655	0.40359	0.075630255	0.10656	28.8293008
Metastasis	KRAS_SIGNALING_UP	182	1.59315	0.28684	0.016806724	0.09281	26.91051697
Inflammation	LYMPHOCYTE_DIFFERENTIATION	179	2.01852	0.29419	0	0.06981	25.2244697
Inflammation	ALLOGRAFT_REJECTION	186	1.62	0.485	0.105	0.08315	25.05387573
Inflammation	TNFA_SIGNALING_VIA_NFKB	197	1.61503	0.33709	0.047325104	0.06465	20.57029332
Inflammation	INFLAMMATORY_RESPONSE	186	1.66245	0.76161	0.043841336	0.06179	19.10768228
Metastasis	IL2_STAT5_SIGNALING	191	1.41526	0.41657	0.07959183	0.06868	18.56527928
Metastasis	APICAL_JUNCTION	184	1.2185	0.7103	0.18292683	0.05574	12.4972619
Inflammation	T_CELL_DIFFERENTIATION	114	2.04165	0.33226	0.002020202	0.03301	7.684114397
Inflammation	COAGULATION	108	1.21138	0.6535	0.24485597	0.05033	6.584123092
Inflammation	LEUKOCYTE_PROLIFERATION	68	1.98195	0.35688	0.00203252	0.02902	3.911450876
Inflammation	IL6_JAK_STAT3_SIGNALING	80	1.47301	0.35848	0.09445585	0.02295	2.704315777
Inflammation	THYMOCYTE_AGGREGATION	41	2.09206	0.34731	0	0.01721	1.475770145

Metabolism	ISOPRENOID_BINDING	20	1.93921	0.34886	0	0.0183	0.70968355
Metastasis	APICAL_SURFACE	41	1.39705	0.39514	0.07581967	0.0094	0.538509757
Inflammation	MAST_CELL_ACTIVATION	15	1.97202	0.32122	0	0.00662	0.19572358

NES: Normalized enrichment score; GeneRatio = enrichment gene count/total gene count; Enrichment Ratio = NES x GeneRatio

Table S5. MiRTarBase and Reactome were analyzed with the 8-miRNA signature.

Function	Pathway name	Entities found	Entities total	Entities ratio	Entities p-value	Entities FDR
Immune system	Interleukin-4 and Interleukin-13 signaling	21	211	0.014906394	5.64E-08	7.73E-05
Cellular response	Oncogene-induced senescence	13	42	0.002967149	4.76E-06	0.003262839
Gene expression	RUNX3 regulates RUNX1-mediated transcription	3	4	2.83E-04	2.65E-05	0.012106852
Disease	TGFBR1 KD mutants in cancer	3	6	4.24E-04	8.76E-05	0.029961642
Disease	Loss of function of TGFBR1 in cancer	3	7	4.95E-04	1.38E-04	0.037732025
Gene expression	Small interfering RNA (siRNA) biogenesis	3	9	6.36E-04	2.87E-04	0.065387672
Cellular response	Cellular senescence	21	198	0.01398799	6.56E-04	0.102699039
Cell cycle	G1 phase	8	48	0.003391028	6.84E-04	0.102699039
Cell cycle	Cyclin D associated events in G1	8	48	0.003391028	6.84E-04	0.102699039
Disease	SMAD4 MH2 domain mutants in cancer	2	3	2.12E-04	8.28E-04	0.102699039
Disease	Loss of function of SMAD4 in cancer	2	3	2.12E-04	8.28E-04	0.102699039
Cellular response	Oxidative stress-induced senescence	13	114	0.008053691	0.00168596	0.179245562
Cellular response	Senescence-associated secretory phenotype	8	89	0.006287531	0.001825798	0.179245562
Signal transduction	ERBB2 regulates cell motility	4	19	0.001342282	0.001847892	0.179245562

Gene expression	Transcriptional regulation by MECP2	15	100	0.007064641	0.00218222	0.198581976
Signal transduction	Pre-NOTCH transcription and translation	10	89	0.006287531	0.002535926	0.215553748
Gene expression	Transcriptional regulation by RUNX3	15	118	0.008336277	0.003617939	0.279122234
Signal transduction	Downregulation of ERBB4 signaling	3	10	7.06E-04	0.003672661	0.279122234
Signal transduction	GRB2 events in ERBB2 signaling	4	20	0.001412928	0.004010608	0.288763746
Gene expression	Posttranscriptional silencing by small RNAs	2	7	4.95E-04	0.004348557	0.29570188
Gene expression	RUNX3 regulates BCL2L11 (BIM) transcription	4	6	4.24E-04	0.005365206	0.3421185
Signal transduction	Regulation of PTEN mRNA translation	4	29	0.002048746	0.005746118	0.3421185
Signal transduction	Regulation of PTEN gene transcription	8	70	0.004945249	0.005798619	0.3421185
Gene expression	FOXO-mediated transcription of cell cycle genes	8	27	0.001907453	0.006153079	0.350725501
Gene expression	MECP2 regulates transcription factors	4	10	7.06E-04	0.006561133	0.354301187

Table S6. The clinicopathological characteristics of TNBC patients in the GSE40049, GSE19783 and E-MTAB-1989 datasets for the testing study.

Dataset	GSE40049	GSE19783	E-MTAB-1989
**Number**			
**TNBC**	24	18	18
**Normal**	14	0	0
**Total**	38	18	18
**Age (years)**	55.52	NA	54
**Preservation type**	Fresh tissue	Fresh tissue	FFPE
**Tumor size**		NA	NA
**T1-T2**	23	-	-
**T3-T4**	1	-	-
**Lymph node metastasis**		NA	NA
**Present**	5	-	-
**Absent**	19	-	-
**Number of recurrence events**	7	7	10
**Median recurrence (years)**	5.1	6.8	2.4
**Platform**	Applied Biosystems SOLiD sequencing	Agilent-019118 Human miRNA Microarray 2.0	A-AFFY-184 - Affymetrix GeneChip miRNA 2.0 Array [miRNA-2_0]

FFPE: Formalin-fixed, paraffin-embedded tissue. NA: not available.

Table S7. Area under the ROC curve for seven and eight miRNAs from two public datasets (GSE40049 and GSE19783) between patients with TNBC recurrence and no recurrence.

GSE40049 in the validation set			
**Model set**	**Number**	**miRNA combinations**	**AUC**
Model_10	7	RNR~miR-139+miR-10b+miR-486+miR-107+miR-324+miR-455+miR-146b	0.9062
Model_11	7	RNR~miR-139+miR-10b+miR-486+miR-107+miR-324+miR-455+miR-20a	0.9062
Model_12	7	RNR~miR-139+miR-10b+miR-486+miR-107+miR-324+miR-146b+miR-20a	0.9062
Model_13	7	RNR~miR-139+miR-10b+miR-486+miR-107+miR-455+miR-146b+miR-20a	0.9062
Model_14	7	RNR~miR-139+miR-10b+miR-486+miR-324+miR-455+miR-146b+miR-20a	0.875
Model_15	7	RNR~miR-139+miR-10b+miR-107+miR-324+miR-455+miR-146b+miR-20a	0.75
Model_16	7	RNR~miR-139+miR-486+miR-107+miR-324+miR-455+miR-146b+miR-20a	0.875
Model_17	7	RNR~miR-10b+miR-486+miR-107+miR-324+miR-455+miR-146b+miR-20a	0.8438
Model_18	8	RNR~miR-139+miR-10b+miR-486+miR-107+miR-324+miR-455+miR-146b+miR-20a	0.9062

GSE19783 in the validation set			
**Model set**	**Number**	**miRNA combinations**	**AUC**
Model_19	7	RNR~miR-139+miR-10b+miR-486+miR-107+miR-324+miR-455+miR-146b	0.7922
Model_20	7	RNR~miR-139+miR-10b+miR-486+miR-107+miR-324+miR-455+miR-20a	0.8831
Model_21	7	RNR~miR-139+miR-10b+miR-486+miR-107+miR-324+miR-146b+miR-20a	0.9091
Model_22	7	RNR~miR-139+miR-10b+miR-486+miR-107+miR-	0.8961

		455+miR-146b+miR-20a	
Model_23	7	RNR~miR-139+miR-10b+miR-486+miR-324+miR-455+miR-146b+miR-20a	0.8701
Model_24	7	RNR~miR-139+miR-10b+miR-107+miR-324+miR-455+miR-146b+miR-20a	0.8571
Model_25	7	RNR~miR-139+miR-486+miR-107+miR-324+miR-455+miR-146b+miR-20a	0.8961
Model_26	7	RNR~miR-10b+miR-486+miR-107+miR-324+miR-455+miR-146b+miR-20a	0.8701
Model_27	8	RNR~miR-139+miR-10b+miR-486+miR-107+miR-324+miR-455+miR-146b+miR-20a	0.8961

Figure S1.

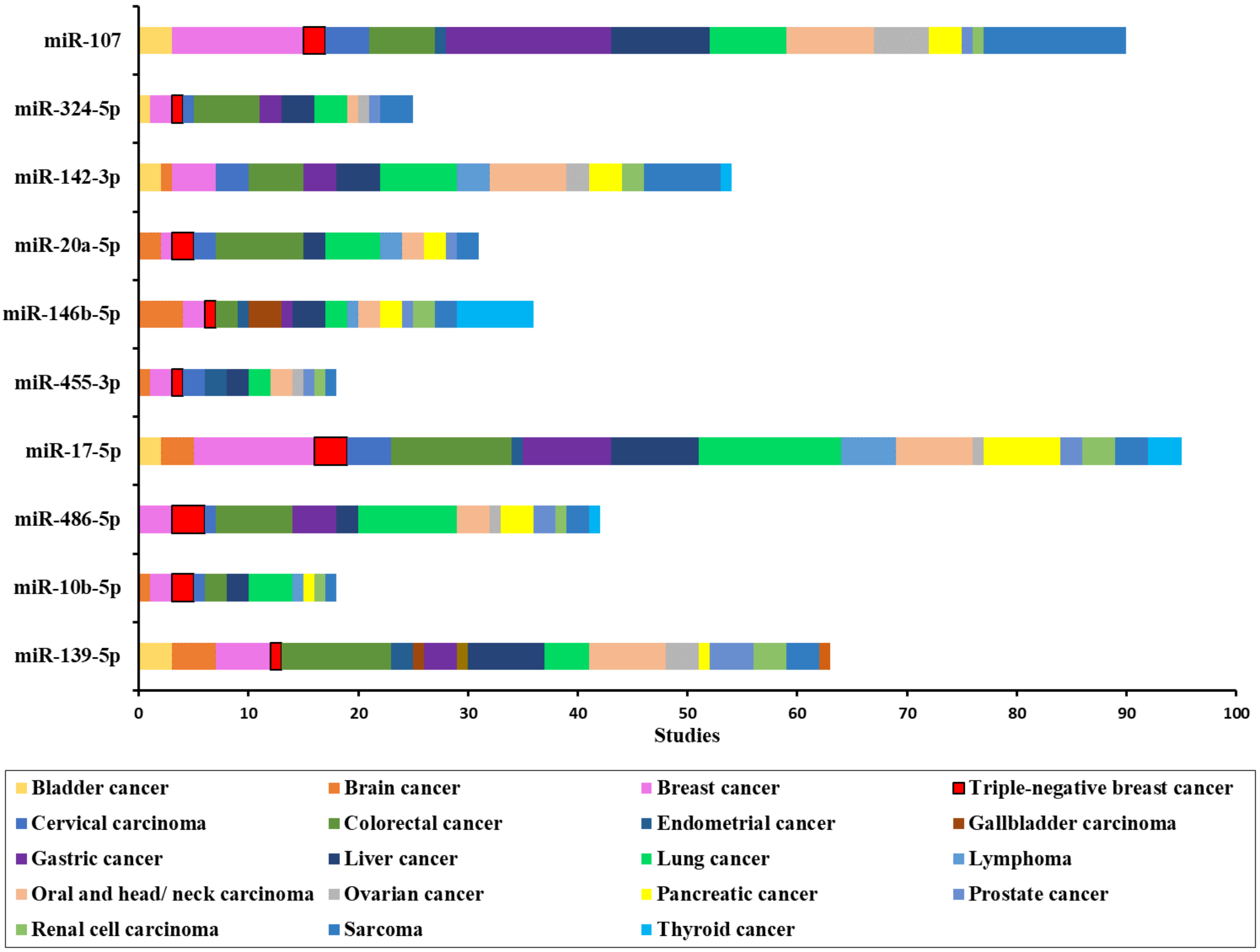

Figure S1. A list of the number of publications on several cancer types associated with these 10 miRNAs.

Figure S2.

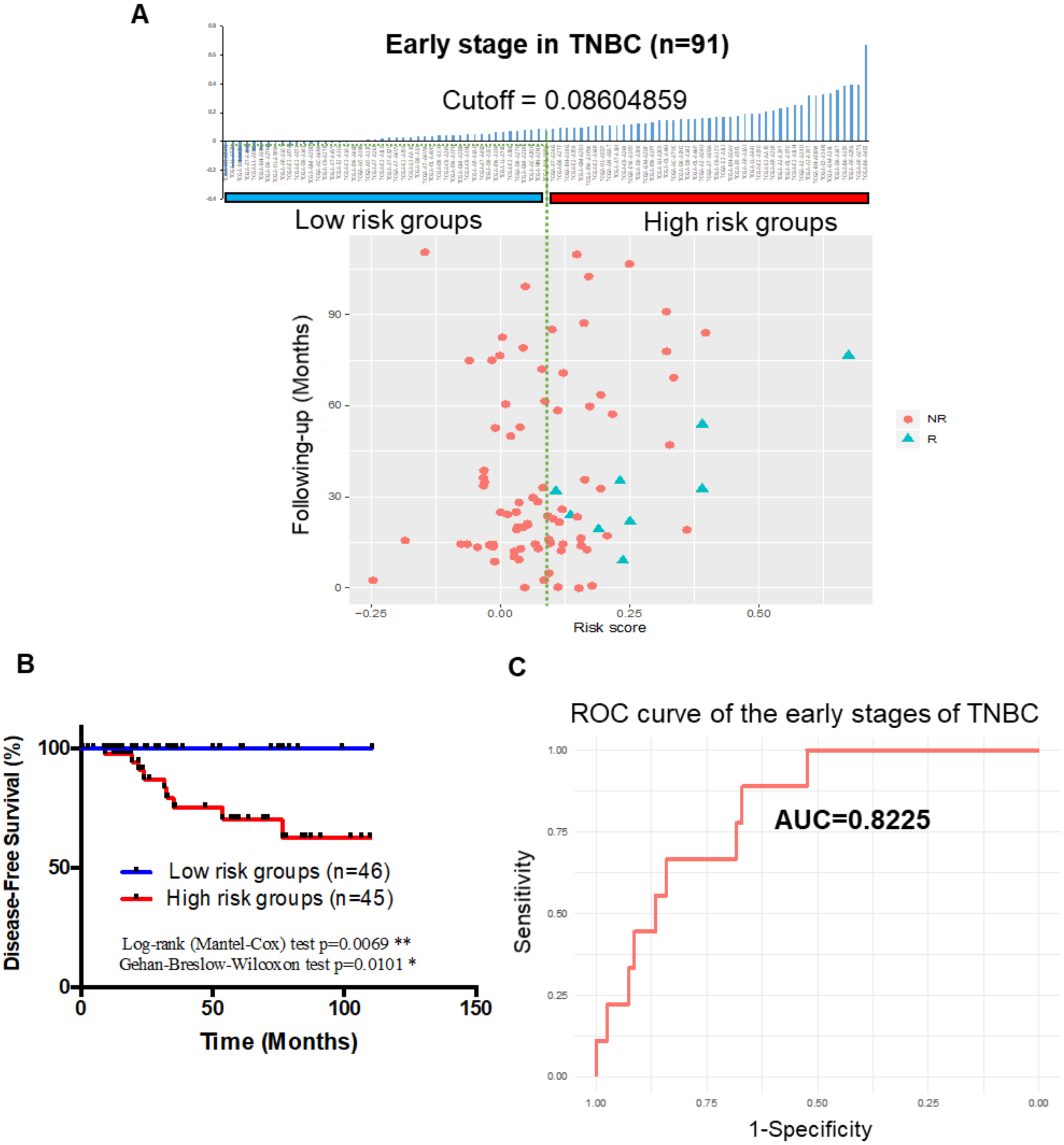

Figure S2. Predictive value of the 8-miRNA signature for 91 patients with early-stage TNBC. (A) The 8-miRNA signature risk score distribution and patient DFS. In the colorgram, the green line represents the median miRNA signature cutoff dividing patients into low- and high-risk groups. **(B)** Kaplan-Meier estimates of DFS in the training set. **(C)** ROC for TNBC recurrence by the 8-miRNA signature between patients with recurrence and without recurrence in the combined or respective miRNAs. The 8 combined miRNAs had the strongest predictive value in the early stage.

Figure S3.

A

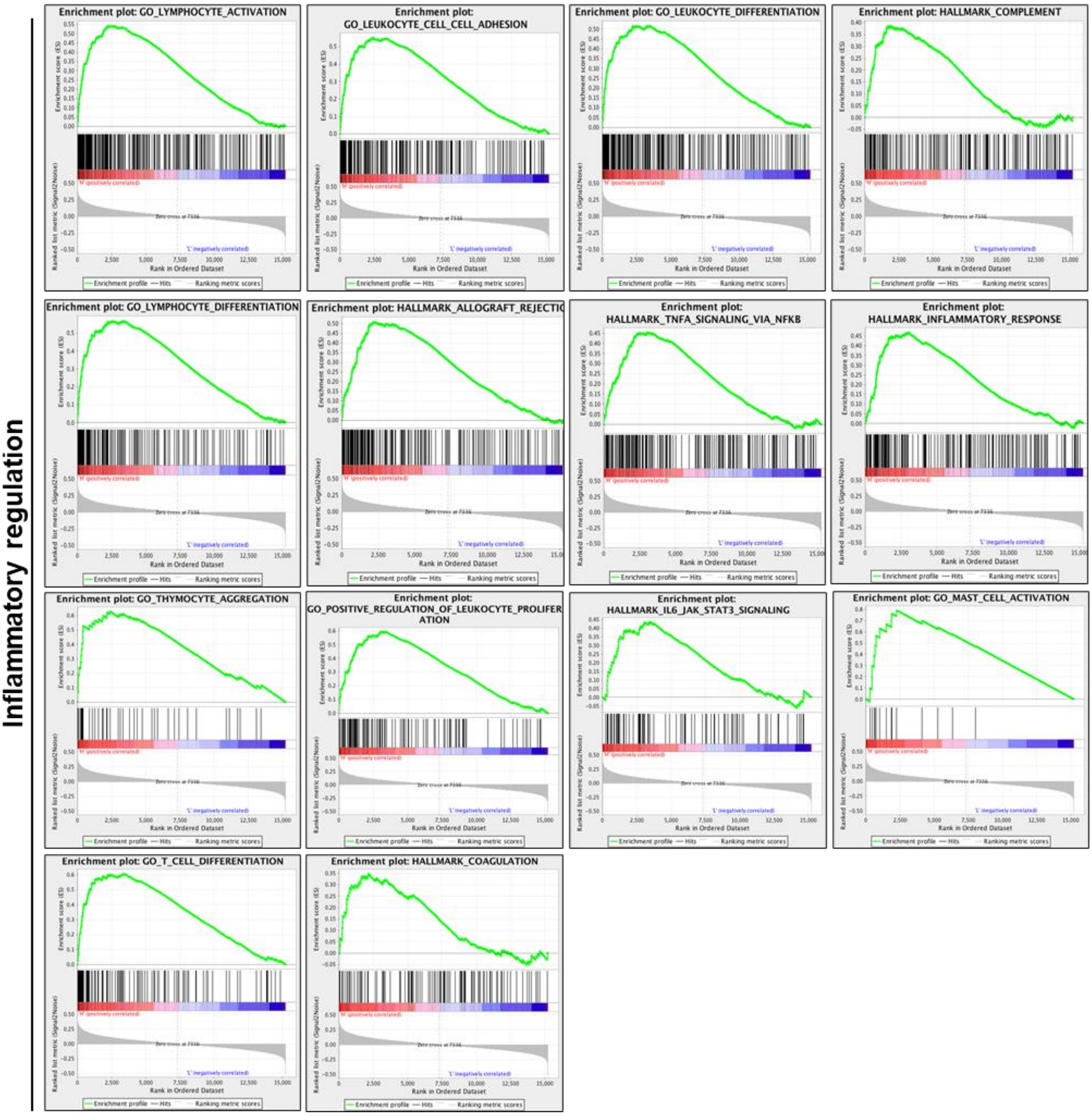
Inflammatory regulation
Enrichment plot: GO_LYMPHOCYTE_ACTIVATION
Enrichment plot: GO_LEUKOCYTE_CELL_CELL_ADHESION
Enrichment plot: GO_LEUKOCYTE_DIFFERENTIATION
Enrichment plot: HALLMARK_COMPLEMENT
Enrichment plot: GO_LYMPHOCYTE_DIFFERENTIATION
Enrichment plot: HALLMARK_ALLOGRAFT_REJECTIC
Enrichment plot: HALLMARK_TNFA_SIGNALING_VIA_NFKB
Enrichment plot: HALLMARK_INFLAMMATORY_RESPONSE
Enrichment plot: GO_THYMOCYTE_AGGREGATION
Enrichment plot: GO_POSITIVE_REGULATION_OF_LEUKOCYTE_PROLIFERATION
Enrichment plot: HALLMARK_IL6_JAK_STAT3_SIGNALING
Enrichment plot: GO_MAST_CELL_ACTIVATION
Enrichment plot: GO_T_CELL_DIFFERENTIATION
Enrichment plot: HALLMARK_COAGULATION

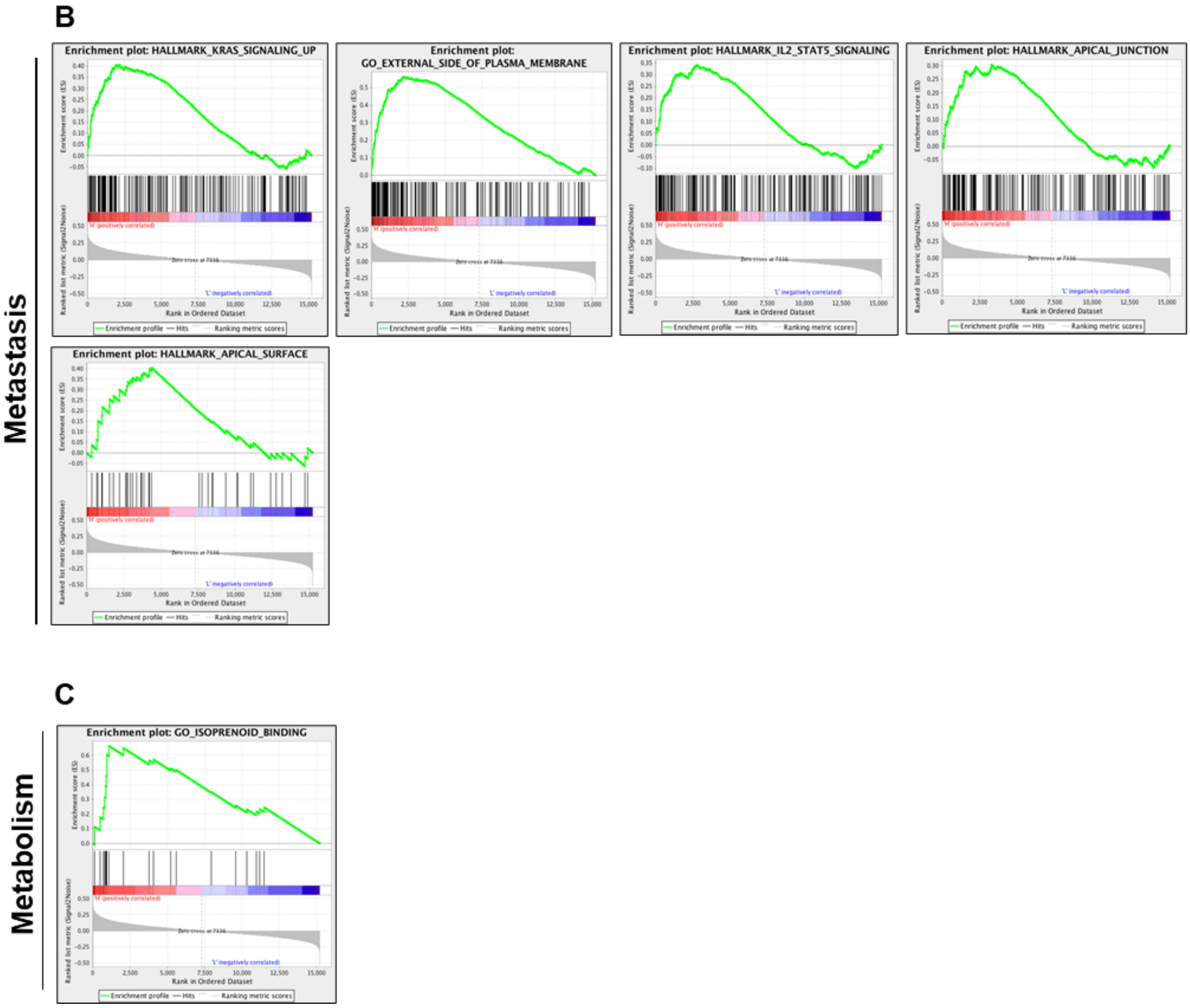

Figure S3. Gene set enrichment analysis (GSEA) comparing the 8-miRNA signature low-risk groups (blue) against the high-risk groups (red) of TNBC patients in TCGA_TNBC dataset. The high-risk groups correlated with inflammatory regulation **(A)** metastasis **(B)** and metabolism **(C)**.

Figure S4.

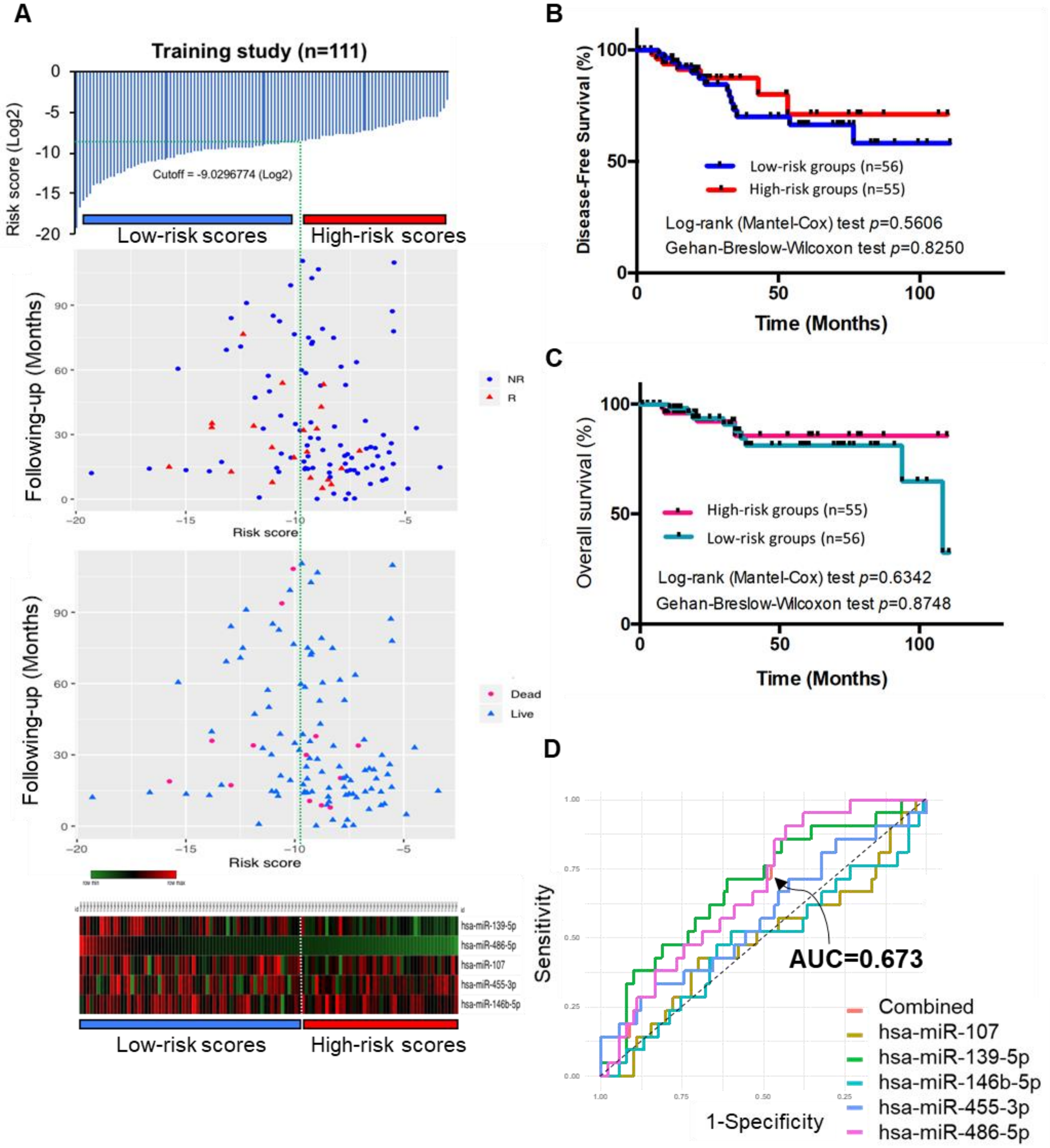

Figure S4. Predictive value of the 5-miRNA signature for 111 TNBC patients. (A) The 5-miRNA signature risk score distribution in the DFS and OS of TNBC patients. The colorgram of 5-miRNA expression profiles of high- and low-risk groups with TNBC. The green line represents the median miRNA signature cutoff dividing patients into low- and high-risk groups. **(B)** Kaplan-

Meier estimates of DFS in the training set. **(C)** Kaplan-Meier estimates of OS in the training set. **(D)** ROC for TNBC recurrence by the miRNA signature between patients with/without recurrence in the combined or respective miRNAs. The predictive value of the 5 combined miRNAs was no different than that of a single miRNA. The *p*-values were calculated using Log-rank and Gehan-Breslow-Wilcoxon tests. R: recurrence; NR: nonrecurrence.

Figure S5.

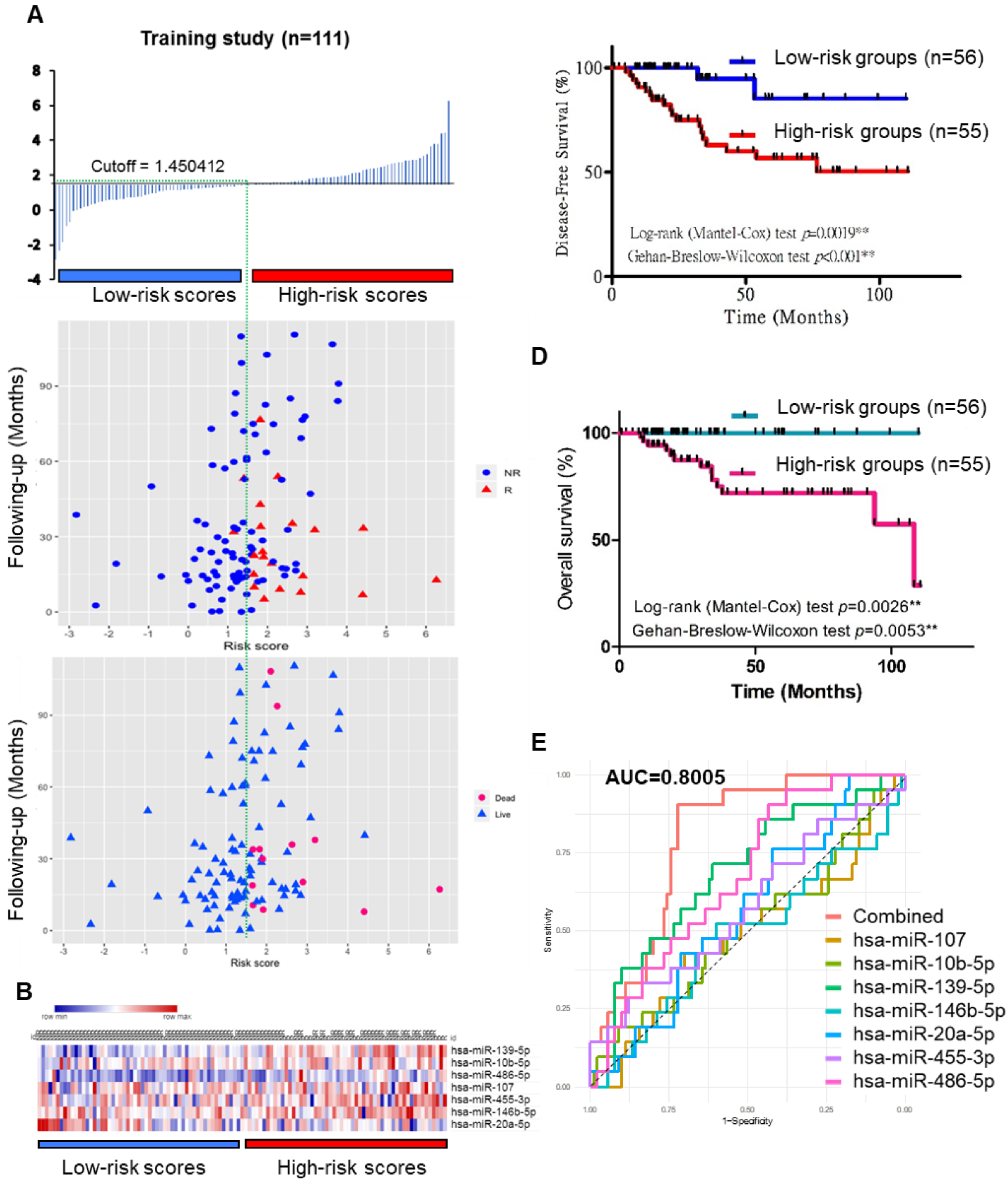

Figure S5. Predictive value of the 7-miRNA signature for 111 TNBC patients. (A) The 7-miRNA signature risk score distribution in DFS and OS of TNBC patients. The colorgram of 7-miRNA expression profiles of high- and low-

risk groups with TNBC. The green line represents the median miRNA signature cutoff dividing patients into the low- and high-risk groups. **(B)** The expression of heatmap in 8 miRNAs for 111 TNBC patients. **(C)** Kaplan-Meier estimates of DFS for the training set. **(D)** Kaplan-Meier estimates of OS for the training set. **(E)** ROC for TNBC recurrence by the miRNA signature between patients with/without recurrence in the combined or respective miRNAs. The 7 combined miRNAs had a stronger predictive value than a single miRNA. The p-values were calculated using Log-rank and Gehan-Breslow-Wilcoxon tests. R: recurrence; NR: nonrecurrence.

Figure S6.

A

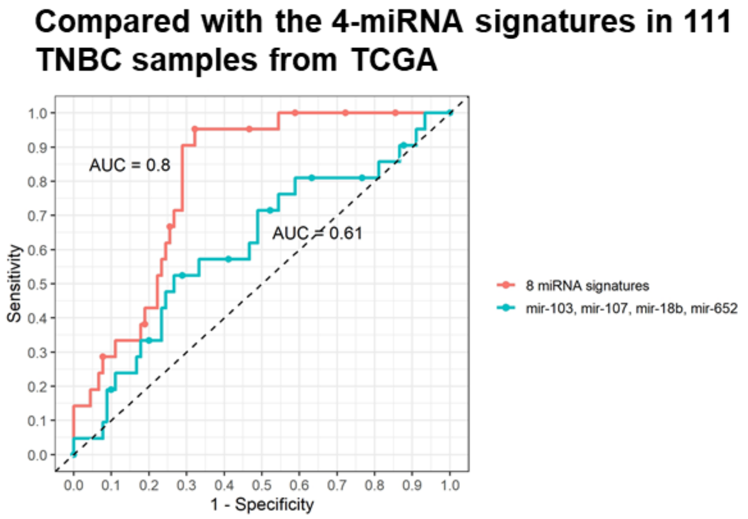

B

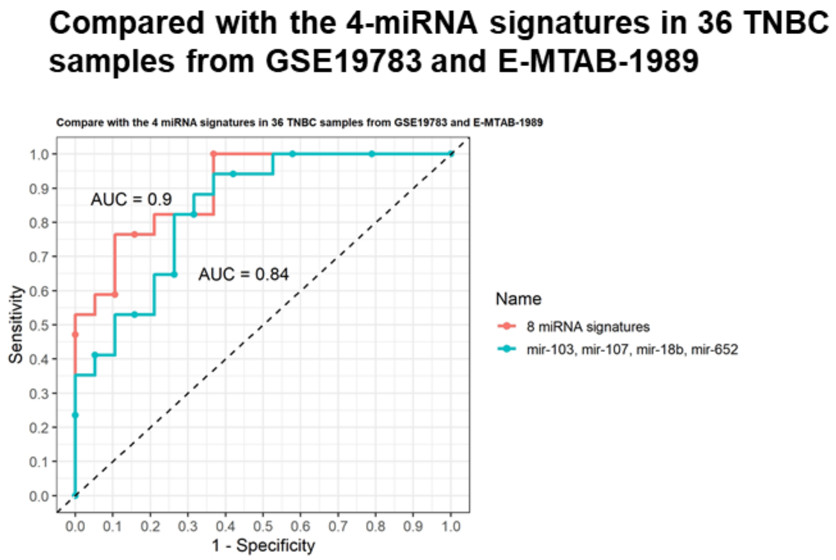

Figure S6. ROC for TNBC patient recurrence by the 4-miRNA signature. The ROC curves generated using the prognosis and expression levels of the 4-miRNA signature were able to discriminate between patients with relapse in 111 patients in TCGA_TNBC **(A)** and 36 patients in the E-MTAB-1989 and GSE19783 datasets **(B)**. The AUC values were 0.61 and 0.84 by the 4-miRNA signature.

Figure S7.

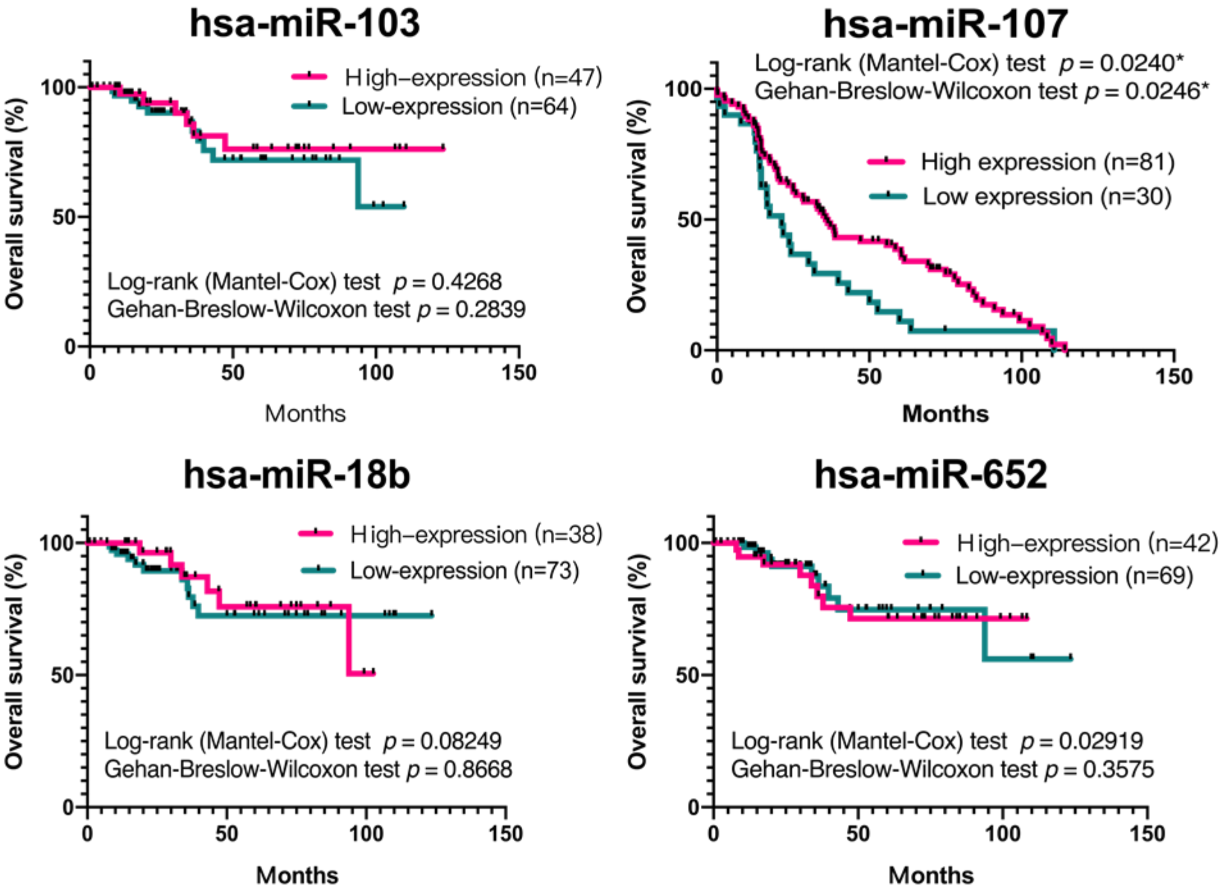

Figure S7. Kaplan-Meier survival analysis estimates the OS of TNBC patients according to the 4-miRNA expression profile. Relative levels of miR-18b, miR-103, miR-107, and miR-652 in 111 patients in TCGA_TNBC with their survival times. The *p*-values were calculated using Log-rank and Gehan-Breslow-Wilcoxon tests. **p*<0.05.

Figure S8.

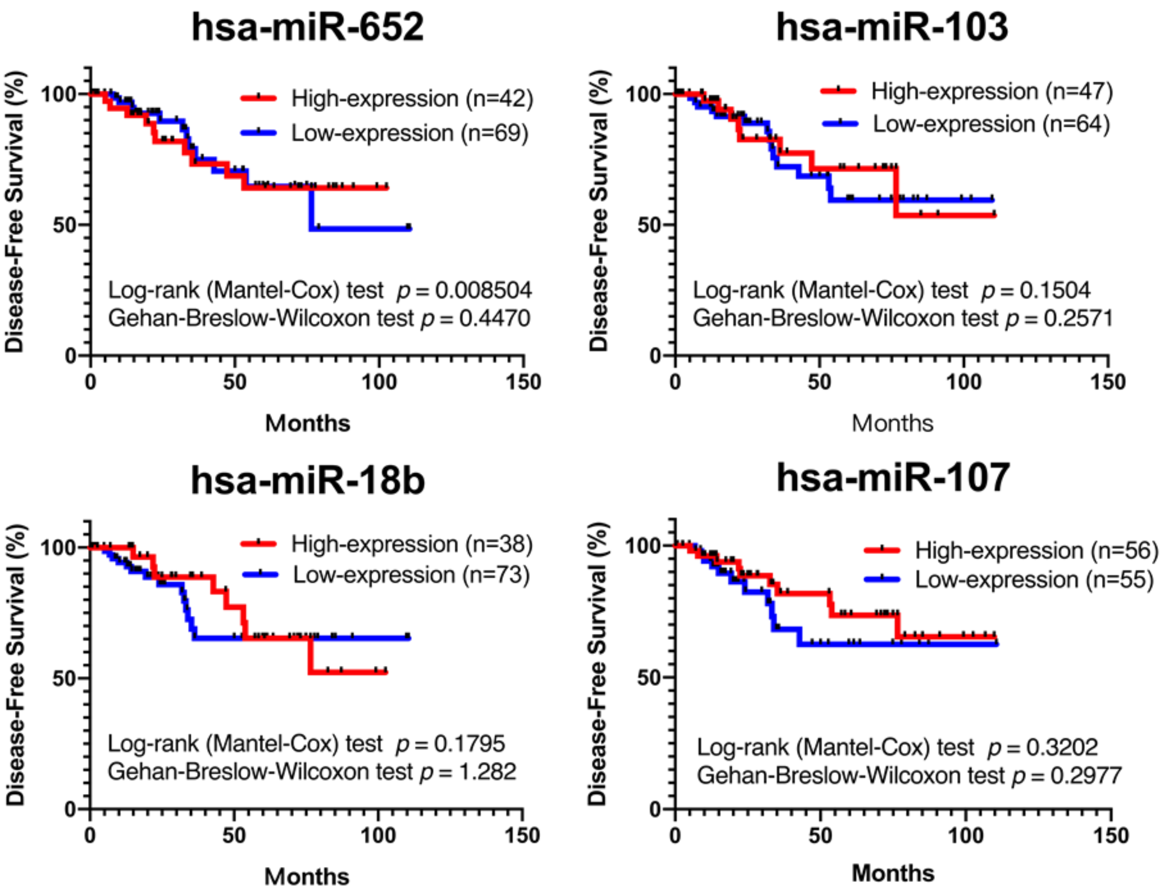

Figure S8. Kaplan-Meier survival analysis estimates DFS of TNBC patients according to the 4-miRNA expression profile. Relative levels of miR-18b, miR-103, miR-107, and miR-652 in 111 patients in TCGA_TNBC with recurrence. The *p*-values were calculated using Log-rank and Gehan-Breslow-Wilcoxon tests.

Figure S9.

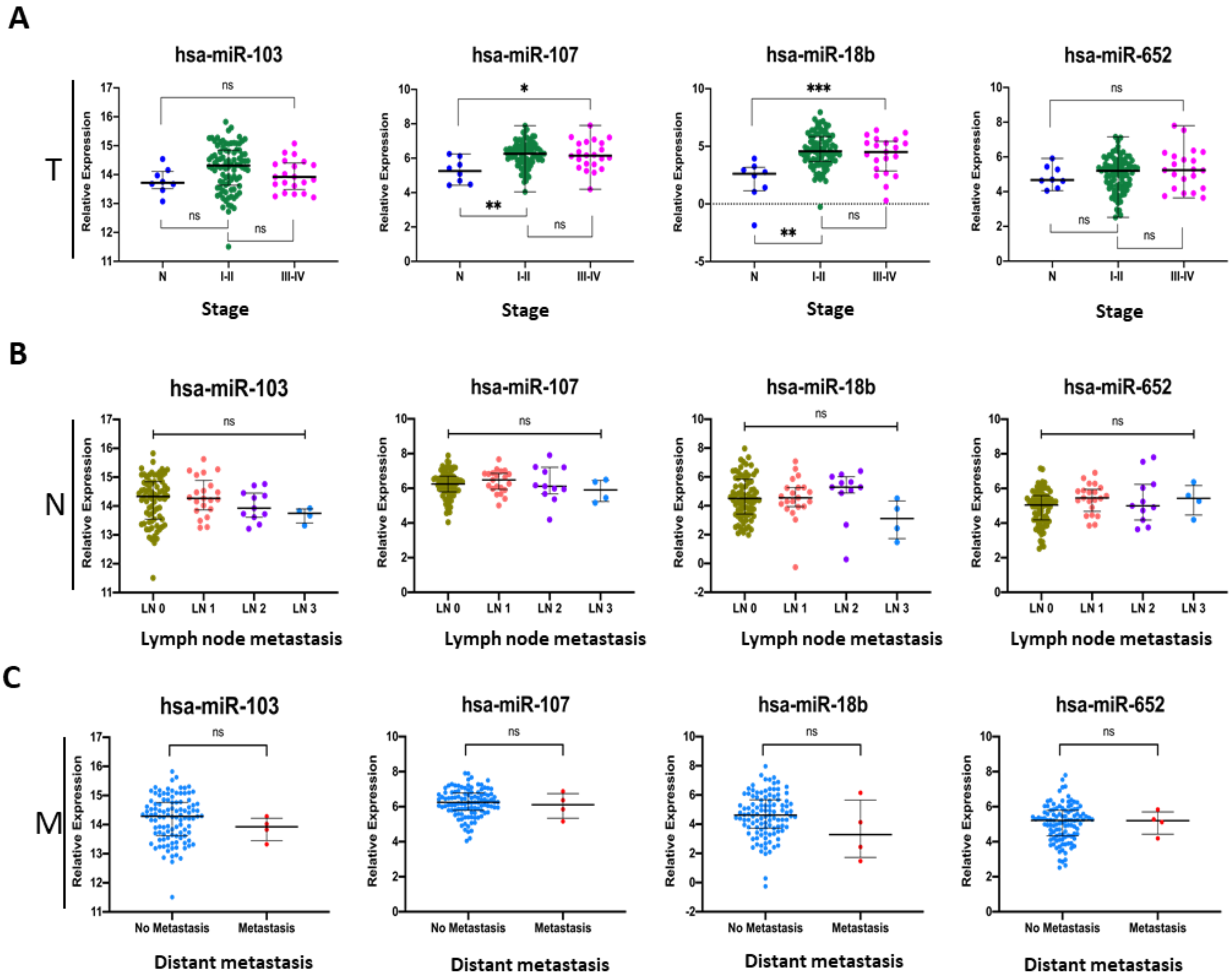

Figure S9. The difference in the 4-miRNA expression profiles in subgroups divided by TNM classification. (A) 111 TNBC patients with 8 N vs. 89 stage I-II vs. 22 stage III-IV. The *p*-values were calculated with TCGA_TNBC. **(B)** The 111 TNBC patients with 74 LN0 vs. 21 LN1 vs. 12 LN2 vs. 4 LN3. The *p*-values were calculated with the Kruskal-Wallis test. **(C)** 111 TNBC patients with 107 no metastasis vs. 4 metastasis. The p-values were calculated using Student's t-test. *p<0.05; **p<0.01; ***p<0.0001; ns is not significant. N: adjacent normal; T: tumor stage; LN: lymph node; M: metastasis.

Figure S10.

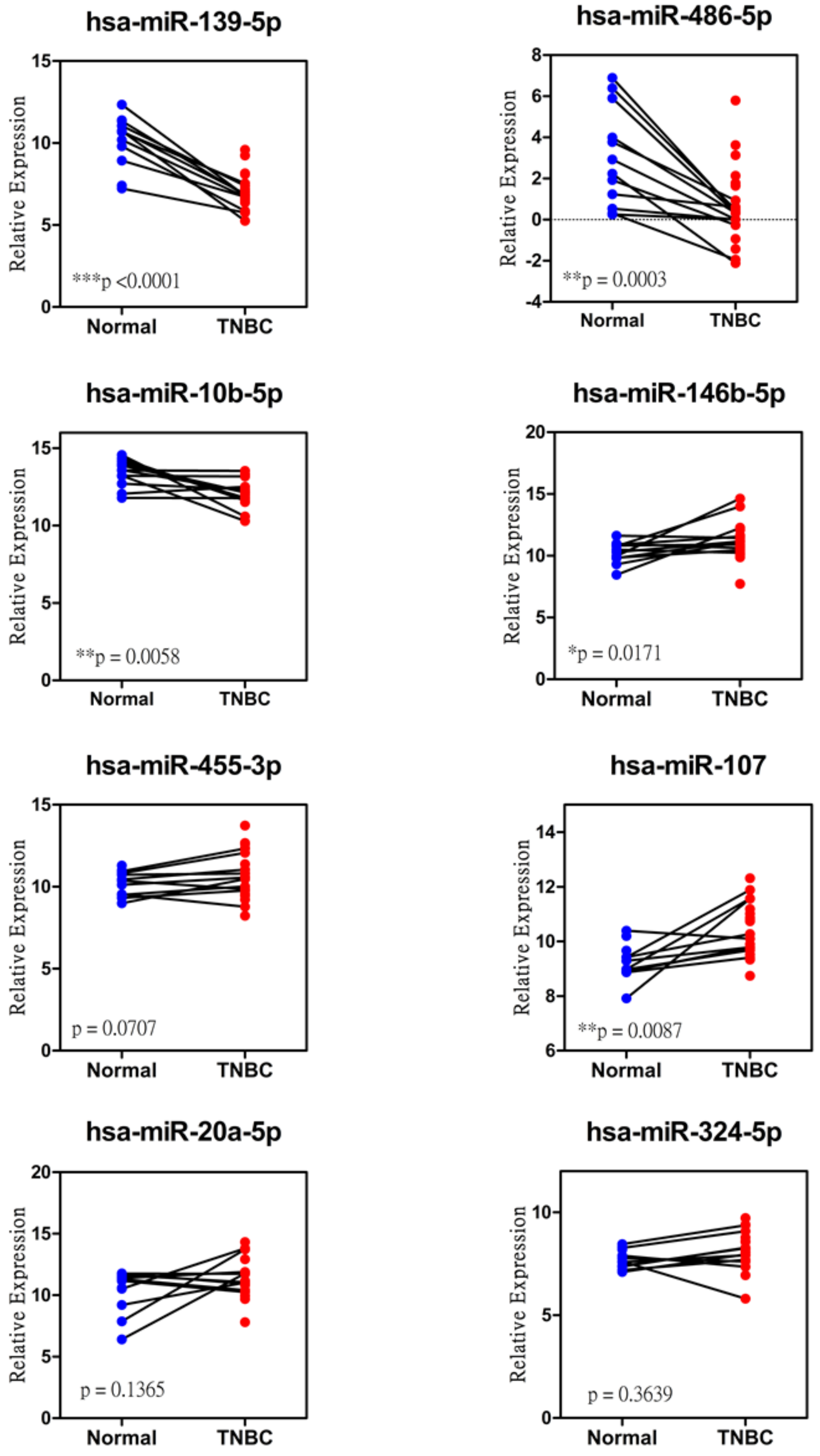

Figure S10. Analysis of the published GSE40049 dataset from clinical data. GSE40049 expression analysis of miR-139-5p, miR-107, miR-146b-5p, miR-142-3p, miR-17-5p, miR-455-3p, miR-324-5p, miR-486-5p, miR-10b-5p and

miR-20a-5p in 24 tumor and 14 adjacent normal tissues. The expression of miR-139-5p, miR-107, miR-146b-5p, miR-10b-5p and miR-486-5p was significantly ($p<0.05$) higher in tumors than in adjacent normal tissues, while the expression of miR-142-3p, miR-17-5p, miR-455-3p, miR-324-5p and miR-20a-5p did not reach statistical significance. The differences between the paired samples were calculated with a Wilcoxon test. *$p<0.05$; **$p<0.01$; ***$p<0.0001$.

Supplementary References

1. Krishnan K, Steptoe AL, Martin HC, Pattabiraman DR, Nones K, Waddell N, et al. miR-139-5p is a regulator of metastatic pathways in breast cancer. RNA (New York, NY). 2013; 19: 1767-80.
2. Yang F, Zhang W, Shen Y, Guan X. Identification of dysregulated microRNAs in triple-negative breast cancer (review). Int J Oncol. 2015; 46: 927-32.
3. Fkih M'hamed I, Privat M, Trimeche M, Penault-Llorca F, Bignon YJ, Kenani A. miR-10b, miR-26a, miR-146a And miR-153 Expression in Triple Negative Vs Non Triple Negative Breast Cancer: Potential Biomarkers. Pathology oncology research : POR. 2017; 23: 815-27.
4. Gupta I, Sareyeldin RM, Al-Hashimi I, Al-Thawadi HA, Al Farsi H, Vranic S, et al. Triple Negative Breast Cancer Profile, from Gene to microRNA, in Relation to Ethnicity. Cancers (Basel). 2019; 11.
5. Ouyang M, Li Y, Ye S, Ma J, Lu L, Lv W, et al. MicroRNA profiling implies new markers of chemoresistance of triple-negative breast cancer. PloS one. 2014; 9: e96228.
6. Turashvili G, Lightbody ED, Tyryshkin K, SenGupta SK, Elliott BE, Madarnas Y, et al. Novel prognostic and predictive microRNA targets for triple-negative breast cancer. FASEB J. 2018: fj201800120R.
7. Abdallah R, Youness R, El Meckawy N, El Sebaaei A, Abdelmotaal A, Assal R. 88P Crosstalk between hesperetin and miR-486-5p in triple-negative breast cancer (TNBC): An approach towards precision medicine. Annals of Oncology. 2018; 29: mdy314. 028.
8. Abdallah R, Youness R, El Meckawy N, El Sebaei A, Abdelmotaal A, Assal R. Paradoxical effects of miR-486-5p on the oncogenic and immunogenic profiles in triple negative breast cancer (TNBC). European Journal of Cancer. 2018; 92: S123.
9. Elkhouly A, Youness R, Gad MJAoO. 172P miR-486-5p counteracts the shedding of MICA/B and CD155 immune-ligands in TNBC patients. 2019; 30: mdz450. 009.
10. Li XY, Luo QF, Wei CK, Li DF, Li J, Fang L. MiRNA-107 inhibits proliferation and migration by targeting CDK8 in breast cancer. International journal of clinical and experimental medicine. 2014; 7: 32-40.
11. Luo Z, Zheng Y, Zhang W. Pleiotropic functions of miR107 in cancer networks. OncoTargets and therapy. 2018; 11: 4113-24.
12. Shen S, Sun Q, Liang Z, Cui X, Ren X, Chen H, et al. A prognostic model of triple-negative breast cancer based on miR-27b-3p and node status. PLoS One. 2014; 9:

e100664.
13. Zhang L, Ma P, Sun LM, Han YC, Li BL, Mi XY, et al. MiR-107 down-regulates SIAH1 expression in human breast cancer cells and silencing of miR-107 inhibits tumor growth in a nude mouse model of triple-negative breast cancer. Mol Carcinog. 2016; 55: 768-77.
14. Li Z, Meng Q, Pan A, Wu X, Cui J, Wang Y, et al. MicroRNA-455-3p promotes invasion and migration in triple negative breast cancer by targeting tumor suppressor EI24. Oncotarget. 2017; 8: 19455-66.
15. Garcia AI, Buisson M, Bertrand P, Rimokh R, Rouleau E, Lopez BS, et al. Down-regulation of BRCA1 expression by miR-146a and miR-146b-5p in triple negative sporadic breast cancers. EMBO molecular medicine. 2011; 3: 279-90.
16. Jin L, Lim M, Zhao S, Sano Y, Simone BA, Savage JE, et al. The metastatic potential of triple-negative breast cancer is decreased via caloric restriction-mediated reduction of the miR-17~92 cluster. Breast cancer research and treatment. 2014; 146: 41-50.
17. Li X, Wu B, Chen L, Ju Y, Li C, Meng S. Urokinase-type plasminogen activator receptor inhibits apoptosis in triple-negative breast cancer through miR-17/20a suppression of death receptors 4 and 5. Oncotarget. 2017; 8: 88645-57.
18. El Majzoub R, Fayyad-Kazan M, Nasr El Dine A, Makki R, Hamade E, Gree R, et al. A thiosemicarbazone derivative induces triple negative breast cancer cell apoptosis: possible role of miRNA-125a-5p and miRNA-181a-5p. Genes & genomics. 2019; 41: 1431-43.
